# Bacterial secretion system skews the fate of *Legionella*-containing vacuoles towards LC3-associated phagocytosis

**DOI:** 10.1038/srep44795

**Published:** 2017-03-20

**Authors:** Andree Hubber, Tomoko Kubori, Cevayir Coban, Takeshi Matsuzawa, Michinaga Ogawa, Tsuyoshi Kawabata, Tamotsu Yoshimori, Hiroki Nagai

**Affiliations:** 1 Research Institute for Microbial Diseases, Osaka University, 3-1 Yamadaoka, Suita, Osaka 565-0871, Japan; 2 Laboratory of Malaria Immunology, Immunology Frontier Research Centre, Osaka University, 3-1 Yamadaoka, Suita, Osaka 565-0871, Japan; 3 Graduate School of Life and Environmental Sciences, Osaka Prefecture University, Rinku Ohrai Kita 1-58, Izumisano, Osaka 598-8531, Japan; 4 Department of Bacteriology I, National Institute of Infectious Diseases, Toyama 1-23-1, Shinjuku-ku, Tokyo 162-8640, Japan; 5 Department of Genetics, Osaka University Graduate School of Medicine, 2-2 Yamadaoka, Suita, Osaka 565-0871, Japan

## Abstract

The evolutionarily conserved processes of endosome-lysosome maturation and macroautophagy are established mechanisms that limit survival of intracellular bacteria. Similarly, another emerging mechanism is LC3-associated phagocytosis (LAP). Here we report that an intracellular vacuolar pathogen, *Legionella dumoffii*, is specifically targeted by LAP over classical endocytic maturation and macroautophagy pathways. Upon infection, the majority of *L. dumoffii* resides in ER-like vacuoles and replicate within this niche, which involves inhibition of classical endosomal maturation. The establishment of the replicative niche requires the bacterial Dot/Icm type IV secretion system (T4SS). Intriguingly, the remaining subset of *L. dumoffii* transiently acquires LC3 to *L. dumoffii*-containing vacuoles in a Dot/Icm T4SS-dependent manner. The LC3-decorated vacuoles are bound by an apparently undamaged single membrane, and fail to associate with the molecules implicated in selective autophagy, such as ubiquitin or adaptors. The process requires toll-like receptor 2, Rubicon, diacylglycerol signaling and downstream NADPH oxidases, whereas ULK1 kinase is dispensable. Together, we have discovered an intracellular pathogen, the survival of which in infected cells is limited predominantly by LAP. The results suggest that *L. dumoffii* is a valuable model organism for examining the mechanistic details of LAP, particularly induced by bacterial infection.

*Legionella* species are opportunistic human pathogens that are normally found within phagocytic protozoa. Infection of humans occurs by incidental inhalation and replication of the organism within alveolar macrophages. This results in an atypical pneumonia termed Legionnaires’ disease. *Legionella* survive in human cells by manipulating host pathways conserved between simple eukaryotes and humans^1^. To subvert host cellular processes, the Dot/Icm type IV secretion system (T4SS) delivers bacterial proteins, termed effectors, into the cytosol of host cells^2^,^3^,^4^.

Although *Legionella pneumophila* accounts for the majority of diagnosed Legionnaires’ disease worldwide, at least 25 different *Legionella* species have been found in patients^5^,^6^. This includes the largely uncharacterized pathogen *Legionella dumoffii*^7^. Why *L. pneumophila* seems to dominantly cause the disease is unknown, though an O-antigen LPS modification is involved^8^,^9^. Dot/Icm T4SSs are required for intracellular replication of *L. pneumophila*^10^,^11^, *L. longbeachae*^12^ and *L. oakridgensis*^13^. The molecular strategies for intracellular survival of *L. pneumophila* have been well examined. Key hallmarks of intracellular *L. pneumophila* replication include association with mitochondria, remodeling of the vacuole into an endoplasmic reticulum (ER)-like compartment, decoration of *L. pneumophila*-containing vacuoles with ubiquitin and modulation of vacuolar phosphoinositides^4^,^14^. *L. pneumophila* also avoids trafficking and degradation within lysosomal compartments by subverting both canonical endocytic maturation and autophagy^15^,^16^,^17^,^18^.

Autophagy is a regulated and fundamental process in eukaryotic cells that acts to degrade and recycle cytoplasmic contents^19^,^20^. It involves at least 30 Atg proteins^21^ and many accessory proteins that govern substrate-targeting in selective autophagy^22^,^23^. The selective autophagic-process that removes bacteria is known as xenophagy, but herein we use autophagy for simplicity. Degradation occurs in terminal membrane-bound compartments termed autophagolysosomes. The process begins with formation of double-membrane autophagosomes that sequester components, including the hallmark autophagy marker LC3/Atg8, before fusing with lysosomes^24^. A number of pathogens that replicate inside membrane-bound compartments, including *Chlamydia, Mycobacterium tuberculosis*, and *Salmonella enterica* serovar Typhimurium (*S*. Typhimurium), gain the autophagy marker LC3 after invasion^25^,^26^,^27^.

Pathogens utilize diverse strategies and virulence factors to escape or subvert autophagy^28^,^29^. Pathogens such as *Coxiella burnetii* actually hijack autophagy and replicate in LC3-positive compartments^30^,^31^. Autophagy has been shown to be dispensable for *L. pneumophila* replication in the protozoan host *Dictyostelium discoideum*^32^. ATG9- knockout data in *D. discoideum* suggests autophagy might restrict *L. pneumophila* in host cells^33^. The potential for Atg machinery to limit *L. pneumophila* is also supported by Atg5-dependent inhibition of *L. pneumophila* replication in normally permissive macrophages following induction of metabolic starvation-triggered autophagy^34^. However, *L. pneumophila* is known to employ multiple strategies to interfere with autophagy. The effector RavZ irreversibly deconjugates LC3 and thus blocks autophagy^17^,^35^. The effector Spl was reported to inhibit autophagy by unknown mechanism^36^.

Key autophagy research has included examination of the contributing mechanisms and molecular triggers. At least six distinct processes or molecules have been found to target bacterial pathogens for decoration with LC3, including (1) innate immune Nod-like receptors^37^, (2) targeting of ubiquitin-modifications associated with the pathogen by various adaptor molecules that also bind to LC3^38^,^39^,^40^,^41^, (3) sensing of damaged vacuolar membranes by galectin-8 and autophagy activation via the adaptor NDP52^42^, (4) Tecpr1^43^, (5) a *Drosophila* peptidoglycan-recognition-protein^44^, and (6) activation of diacylglycerol (DAG) signaling^45^,^46^,^47^.

An alternative process for acquiring LC3 that is termed LC3-associated phagocytosis (LAP) has also been described for dead and entotic cells, zymosan (dead yeast) and a few bacteria^48^,^49^. A hallmark of this process is the formation of an LC3-positive phagosome with a single membrane^50^. Separately, single membrane phagosomes and association with LC3 have been described for the normally cytosolic pathogens *Burkholderia pseudomallei* and *Mycobacterium marinum*^51^,^52^,^53^. Compartments containing *M. marinum*^54^, *Shigella flexneri*^55^, *Listeria monocytogenes*^46^, and *Yersinia pseudotuberculosis*^56^,^57^ have been confirmed, at least in part, as LC3-positive single-membrane compartments. For the two latter organisms, the bacteria somehow replicate within this compartment. Restriction of intracellular bacterial replication by LAP has not been reported, though a survival defect was reported for *B. pseudomallei* in Beclin 1 siRNA treated cells^58^.

Here we profiled *L. dumoffii*-containing vacuoles (LdCVs) and found that the process of LAP conjugates LC3 to LdCVs, which results in bacterial degradation.

## Results

### *L. dumoffii* avoids canonical endocytic maturation and replicates inside an ER-like compartment

**Figure 1:**
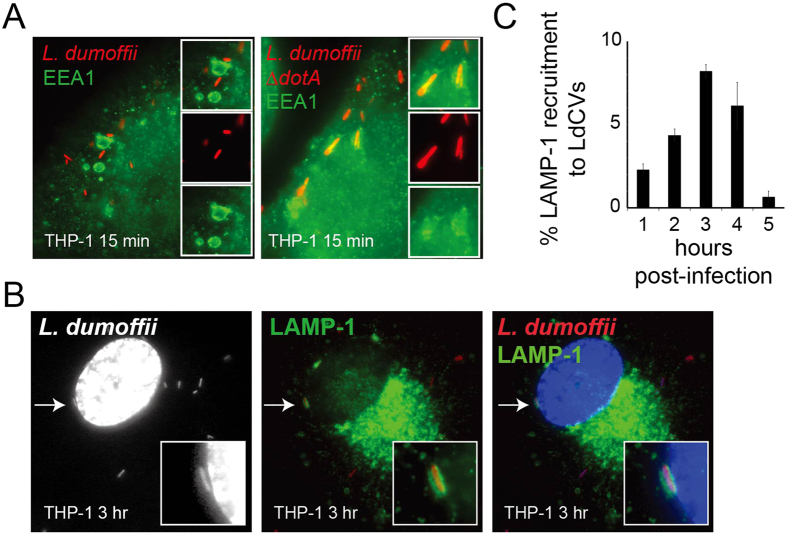
Localization of endosomal markers on LdCVs. (**A**) *L. dumoffii*-containing vacuoles (LdCVs) preclude an early endosomal marker the early-endosomal antigen 1 (EEA1). Association of EEA1 with LdCVs was examined in THP-1 cells at 15 min post-infection. EEA1 was visualized by indirect immunofluorescence using the anti-EEA antibody (Sigma-Aldrich #E4156). Cells were infected with *L. dumoffii* strains constitutively expressing mCherry. (**B**) Some LdCVs acquire late endosomal and lysosomal marker LAMP-1 in THP-1 cells at 3 hours post infection (arrows and insets). LAMP-1 association with mCherry-expressing *L. dumoffii* was examined using indirect immunofluorescence with H4A3 (Santa Cruz #sc-20011). (**C**) Quantification of LAMP-1 recruitment to LdCVs at indicated time points post infection in THP-1 cells. Data is averaged values ± standard error of the mean (SEM) from two independent experiments, each performed in triplicate with 100 vacuoles scored for each sample.

*L. pneumophila* avoids endocytic maturation and creates an ER-like niche. To assess whether *L. dumoffii* also interferes with the classical endocytic pathway, we examined acquisition of the tethering protein early endosomal antigen (EEA) 1, and the lysosomal marker LAMP-1. These markers have previously been used to examine Dot/Icm-dependent trafficking of *L. pneumophila*^16^,^59^. EEA1 association was not commonly observed on LdCVs in THP-1 cells (Fig. 1A). To examine whether or not this is a Dot/Icm-dependent inhibition we created an *L. dumoffii* mutant lacking the *dotA* gene. The *L. dumoffii* ∆*dotA* strain failed to prevent EEA1 association (Fig. 1A), and was unable to replicate inside host cells (Figure S1A). Consistent with a block in endosomal maturation, LAMP1-association with LdCVs was rarely observed on LdCVs at one hour post-infection. However, we unexpectedly observed delayed acquisition of LAMP1 with ~10% of LdVCs positive at 3 to 4 hours post-infection (Fig. 1B,C).

To test whether LdCVs become ER-associated, we examined phagosomal acquisition of two different markers: mtagRFP-KDEL (ER retention signal) and the ER-resident protein calnexin. On *L. pneumophila*-containing vacuoles (LpCVs), KDEL proteins transiently accumulate prior to maximal calnexin recruitment^60^. Similarly, mtagRFP–KDEL was highly enriched on LdCVs at 90 min post-infection (Figure S1B). Calnexin was not detected on LdCVs at 3 hours, but vacuoles containing replicating *L. dumoffii* were calnexin-positive (Figure S1C).

Taken together, *L. dumoffii* inhibits endosomal maturation and creates an ER-like niche, as previously described for *L. pneumophila*. However, the slow kinetics of maximal LAMP-1 acquisition was unexpected.

### LC3 associates with a subset of LdCVs in a T4SS-dependent manner

**Figure 2:**
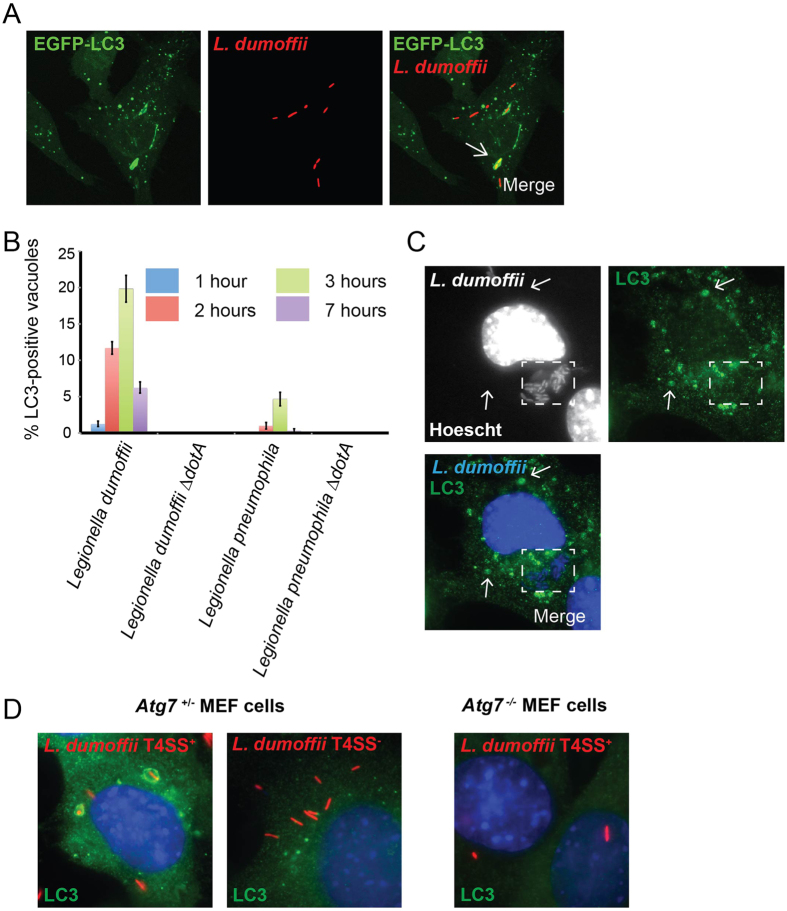
LdCVs become transiently decorated with LC3 in a Dot/Icm-dependent manner. (**A**) Confocal z-stack of RAW cells stably expressing GFP-LC3 after infection with *L. dumoffii* for 3 hours. The representative LC3-positive vacuole is shown by a white arrow. (**B**) Quantification of LC3-association to LdCVs and LpCVs over time in THP-1 cells. Wild-type *L. dumoffii* capable of translocating bacterial effectors elicited LC3-decoration, whereas the translocation-deficient strains (∆*dotA*) did not. (**C**) *L. dumoffiii ∆flaA* in a mouse embryonic fibroblast (MEF) at 16 hours post-infection. Despite the presence of LC3-positive puncta (white arrows), significant association with the bacterial compartment (dotted white square) was not observed. (**D**) Representative images of LC3-association with T4SS^+^ and T4SS^−^ (∆*dotA) L. dumoffii* in *Atg7*^+/−^ MEF cells, and T4SS^+^ *L. dumoffii* in *Atg7*^−/−^ MEF cells. In panels C and D, LC3 was detected by indirect immunofluorescence using anti-LC3 antibody (MBL#PM036). Red-coloured *L. dumoffii* constitutively express mCherry.

In addition to the classical endosome-lysosome pathway, another process that could deliver bacterial pathogens to LAMP-1-positive compartments is autophagy. Thus, we examined whether LdCVs acquire the autophagy marker LC3. In RAW 264.7 cells stably expressing GFP-LC3, GFP-LC3 localization was clearly observed on a minority of LdCVs (Fig. 2A). We then compared the LC3-recruitment phenotype of *L. pneumophila* and *L. dumoffii* in THP-1 cells (Fig. 2B). LpCVs rarely showed LC3 association, but LC3 was associated with up to ~20% of LdCVs. The peak of association was observed at three hours post-infection. LC3 was not observed on mature vacuoles containing replicating *L. dumoffii* (Fig. 2C). Importantly, we found that decoration with LC3 was Dot/Icm-dependent as our ∆*dotA* deletion strain remained LC3-negative (Fig. 2B).

To examine whether LdCV LC3-acqusition involves host Atg machinery we assessed LC3 association to LdCVs in *Atg7*^+/−^ and *Atg7*^−/−^ mouse embryonic fibroblasts (MEFs). In the *Atg7*^−/−^ MEFs LC3-was not observed (Fig. 2D). Thus, this finding suggests that autophagy components engage with *L. dumoffii* in a process that requires the virulence activities of the pathogen.

### LdCVs are devoid of autophagy signaling molecules and adaptors

**Figure 3:**
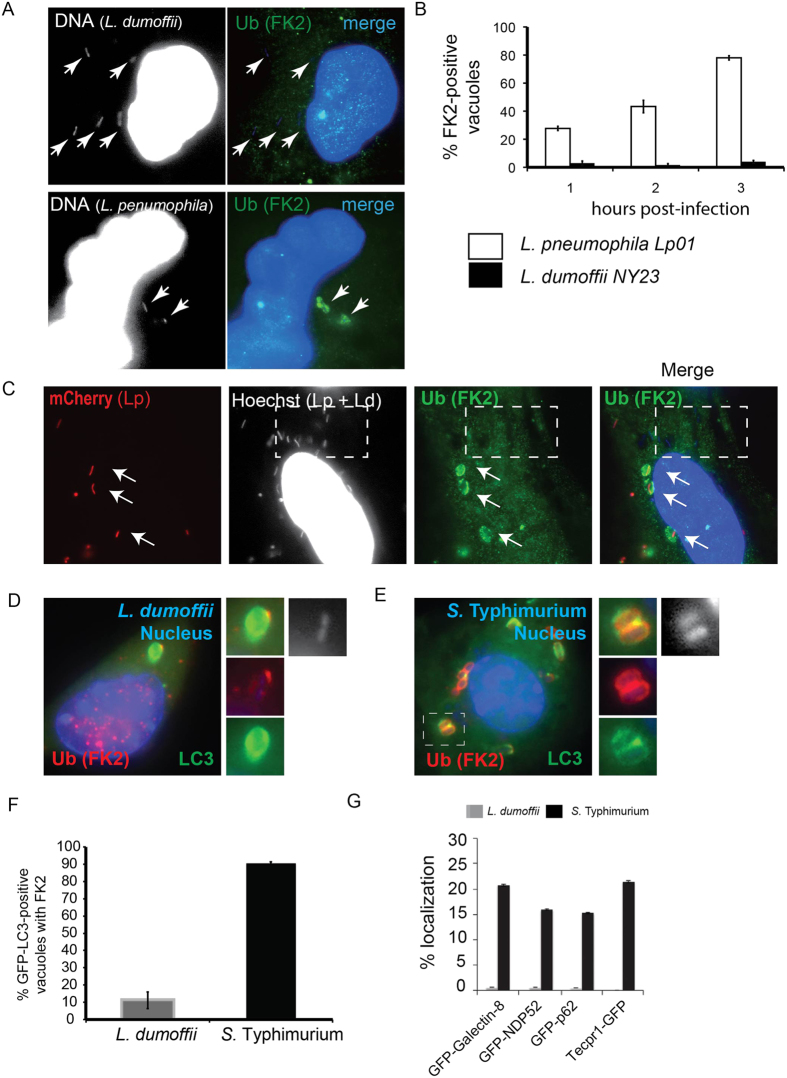
LdCVs become decorated neither with ubiquitin nor with selective-autophagy adaptor proteins. (**A**) Representative micrographs of THP-1 cells infected with *L. dumoffii* or *L. pneumophila* for 3 hours. Nuclear and bacterial DNAs are stained with Hoechst 33342, and ubiquitin is stained using anti-ubiquitin antibody (clone FK2). Arrows describe locations of bacteria. (**B**) Quantification of ubiquitin association with vacuoles containing either *L. pneumophila* or *L. dumoffii* in THP-1 cells. Data is averaged values ± standard error of the mean (SEM) from two independent experiments, each performed in triplicate with 100 vacuoles scored for each sample. (**C**) Representative micrograph of THP-1 cells co-infected with mCherry-expressing *L. pneumophila* and unlabelled *L. dumoffii* at 3 hours post-infection. Ubiquitin staining was performed using anti-ubiquitin (FK2) antibody. The blue (Hoescht 33342) but not red bacteria are *L. dumoffii*. Ubiquitin was commonly associated with LpCVs (arrows), but was absent from LdCVs (white dotted squares). (**D**,**E**) Fluorescent micrographs of RAW264.7 cells stably expressing GFP-LC3 and infected with *L. dumoffii* (**D**) or *S*. Typhimurium (**E**). Ubiquitin staining was performed using anti-ubiquitin (FK2) antibody. Both nuclear and bacterial DNAs are shown in blue (Hoescht 3342). (**F**) Quantification of ubiquitin association with GFP-LC3-positive LdCVs and SCVs in RAW264.7 cells at 3 hours post-infection. Data is average of two independent experiments performed in triplicate. (**G**) Quantification of localization of indicated autophagy adaptor proteins to LdCVs (grey bars) and to SCVs (black bars) in HeLa cells. Data is average from two independent experiments, each performed in triplicate. See Figure S2 for detailed experimental procedure.

We next examined the molecular mechanism behind LC3-decoration of *L. dumoffii*. Recognition of substrates decorated with ubiquitin, a 76-amino acid polypeptide, is a common feature of selective autophagy^61^,^62^, and, to date, all *Legionella* species tested have been shown to reside in vacuoles that are decorated with ubiquitin^63^. Thus, we analyzed whether *L. dumoffii* also resides within a ubiquitin-positive compartment that could contribute towards host recognition by autophagy. To do this, we infected THP-1 cells and assessed ubiquitin-localization using the ubiquitin (clone FK2) antibody, which detects both mono- and poly-ubiquitin conjugates but not free ubiquitin, at various stages of infection. Although LpCVs were decorated with ubiquitin, we did not detect significant localization at any stage of infection with LdCVs (Fig. 3A,B). We also examined ubiquitin-localization in cells co-infected with both *L. pneumophila* (mCherry/Hoechst 33342 double stained in Fig. 3C) and *L. dumoffii* (Hoechst 33342 single stained). Whereas ubiquitin-decoration was consistently observed for LpCVs, LdCVs found in the same cell remained ubiquitin-negative. This result further confirmed that LdCVs are largely devoid of ubiquitin. We also confirmed that LC3-positive LdCVs are largely ubiquitin-negative (Fig. 3D,F). *Salmonella enterica* serovar Typhimurium (*S*. Typhimurium)-containing vacuoles (SCVs), which are established for ubiquitin-mediated recognition by autophagy^64^, were found to be mostly ubiquitin-positive (~90%) (Fig. 3E,F). These results suggest that formation of LC3-positive LdCVs occurs via ubiquitin-independent mechanisms.

We next examined involvement of signaling molecules and adaptor proteins that are commonly found to target pathogens for LC3-decoration. Once again we used *S*. Typhimurium as a positive control because it is reported to recruit the ‘danger’ signal galectin-8 and the LC3-binding adaptors p62, NDP52, and Tecpr-1 on its vacuoles^38^,^39^,^42^,^43^,^65^. Consistently, localization of GFP-galectin-8, GFP-NDP52, GFP-p62 and Tecpr1-GFP was readily observed on SCVs (Figs 3G and S2). In contrast, localization of these constructs was rarely (<2%) observed on LdCVs. Thus, it appears that decoration of *L. dumoffii* with LC3 is largely independent of ubiquitin, galectin-8, and the adapters NDP52, p62 and Tecpr-1.

### LC3-associated LdCVs are single-membrane-bound vacuoles

**Figure 4:**
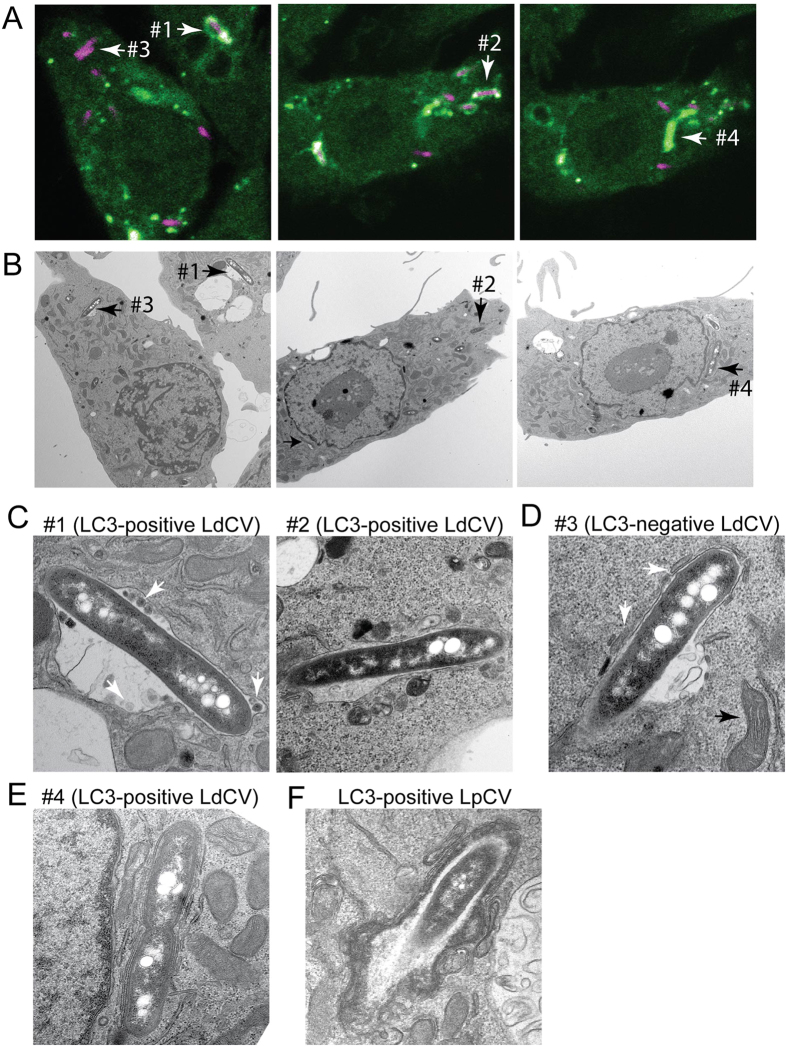
LC3-associated LdCV is bordered by a single membrane. (**A**,**B**) CLEM analysis of LdCVs. RAW264.7 cells stably expressing EYGP-LC3 were cultured on glass bottom dishes (with grids) and then infected with mCherry-expressing *L. dumoffii* for 2.5–3 hours. Cells were fixed and fluorescent images obtained using a confocal microscope (**A**). Specimens were then further examined by transmission electron microscopy (TEM) (rest of all panels). TEM images are of the same field as the fluorescent micrographs (**B**). The representative LdCVs indicated by arrows with number (#1–#4) were further analysed by high-magnification TEM (**C**–**E**). (**C**) High-magnification TEM images of LC3-positive single-membrane-bound LdCVs. Small intracellular vesicles are denoted by white arrows (see text) in TEM images of LdCV #1. (**D**) A representative LC3-negative LdCV was also examined, as shown by LdCV #3. A large black arrow shows nearby mitochondria with internal cristae visible and a white arrow shows vesicles attached to the LdCV. (**E**) The LdCV #4 is a rare (~7%) LC3-positive double-membrane LdCVs, which is consistent with conventional autophagy. (**F**) A micrograph of a rare LC3-positive LpCVs formed in the RAW264.7 GFP-LC3 cell line.

It is emerging that Atg proteins play roles in processes beyond classical autophagy^66^. Indeed, two degradative pathways are associated with the core autophagy marker LC3/Atg8: autophagy and LC3-associated phagocytosis (LAP). Because LAP is not associated with ubiquitinated proteins^47^ and LdCVs lack ubiquitin and common autophagy adaptors, we decided to determine the pathway required for LC3-association. One of major differences between autophagy and LAP is the formation of a double-membrane-bound autophagosome during autophagy versus a single-membrane-bound LAP-phagosome. Thus, we examined the membranes of LC3-positive LdCVs using correlative light and electron microscopy (CLEM) at 2.5 to 3 hours post-infection (Fig. 4). This technique allows confocal and electron- micrographs to be taken of the same cell^57^. We examined 15 separate LC3-positive LdCVs from seven independent dishes. The vast majority of LC3-positive bacteria resided in spacious vacuoles with a single membrane (Fig. 4A–C, LdCVs #1 and #2). Small intracellular vesicles were also observed within the lumen of these enlarged LC3-positive compartments (Fig. 4C, LdCV #1, white arrows). These may be artifacts or perhaps intraluminal vesicles^67^. LC3-negative vacuoles had attached vesicles (Fig. 4D, white arrows) and adjacent mitochondria (Fig. 4D, black arrow), which are both common hallmarks found in EM images of LpCVs^68^,^69^. Only a single double-membrane structure was found associated with an LC3-positive LdCV (Fig. 4A,B,E, #4). We also examined a rare LC3-positive *L. pneumophila* vacuole and observed a multi-membrane structure that is consistent with conventional autophagy (Fig. 4F). These results are consistent with the hypothesis in which a subset of LdCVs is targeted to LAP rather than conventional autophagy.

### TLR2 contributes towards LC3-decoration of LdCVs

**Figure 5:**
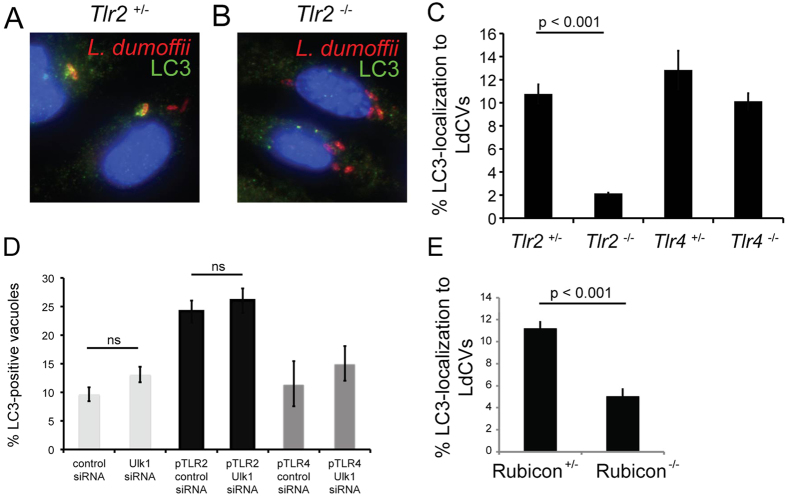
LC3-recruitement to LdCVs is dependent on TLR2 but not Ulk1. (**A**–**C**) Bone marrow-derived macrophages were obtained from the femur and tibia of heterozygous *Tlr2*^+/−^ and *Tlr4*^+/−^ mice and homozygous *Tlr2*^−/−^ and *Tlr4*^−/−^ mice. After differentiation into bone-marrow derived macrophages, cells were infected with mCherry expressing *L. dumoffii* ∆*flaA* for 3 hours. LC3 was detected by indirect immunofluorescence (**A**,**B**) and localization scored in three independent experiments (**C**). (**D**) Quantification of LC3-recruitment in mock, TLR2 or TLR4-expressing HEK293 cells treated with scrambled siRNA or Ulk1 siRNA for 3 days prior to infection with mCherry expressing *L. dumoffii ∆flaA* for 3 hours. Data are the average of three independent experiments, each performed with triplicate wells. (**E**) Bone marrow-derived macrophages were obtained from the femur and tibia of heterozygous Rubicon^+/−^ and homozygous Rubicon^−/−^ mice. *L. dumoffii* infection and LC3 detection was carried out as described for panels A–C.

Because TLRs have been implicated in LAP-activation^50^, we examined the impact of TLR signaling on LC3-acquisition using murine bone-marrow derived macrophages (BMDMs) (Fig. 5). *L. pneumophila* is restricted within C57BL/6J (B6) mice by flagellin-induced Naip5 (Birc1e)/Nlrc4 (IPAF)-dependent Caspase-1-mediated cell death^70^,^71^,^72^,^73^. To avoid possible complications due to murine-specific restriction, we created a mutant *L. dumoffii* strain lacking the flagellin-encoding gene *flaA*. Our *L. dumoffii ∆flaA* strain was capable of robust replication within BMDMs from B6 mice (Figure S3). In BMDMs obtained from TLR2^−/−^ knockout mice, LC3-positive LdCVs were formed at a lower level than the TLR2^+/−^ control (Fig. 5A–C). In comparison, TLR4 signaling was found to be dispensable for LC3-acquisition (Fig. 5C). Thus, these results support the link between TLR2 and LAP, which was previously established using the TLR2 agonist zymosan^50^.

### Ulk1 is dispensable for LC3-decoration of *L. dumoffii*

A mechanistic variable known to distinguish LAP from conventional autophagy is the requirement for the ULK (Ulk1-Atg13-Fip200) complex: Ulk1 (Unc51-like kinase autophagy activating kinase (1) is not required for LAP^50^,^74^,^75^. Conversely, conventional autophagy is Ulk1-dependent. Under non-inducing conditions this protein is functionally inhibited by the mammalian-target of rapamycin (mTOR), but after relief from repression the ULK complex activates formation of the initial isolation membrane^76^. We found that TLR2, but not TLR4, expression in non-immune HEK293 cells allows LdCV enhanced LC3-recruitment (compare control siRNA columns in Fig. 5D). The result on the transfectable cell-line enabled us to analyze the role of Ulk1 using siRNA knockdown. HEK293 cells were co-transfected with TLR2 and siRNA-targeting Ulk1 or scrambled siRNA. Levels of LC3-association with *L. dumoffii* were found to be comparable despite the reduced level of Ulk1 (Figs 5D and S4A,B). To validate the effect of Ulk1 knockdown, in uninfected cells we compared the effect of the autophagy activator rapamycin on LC3-puncta formation following scrambled or Ulk1 siRNA treatment. As expected, the addition of rapamycin to cells treated with scrambled siRNA led to increased LC3 puncta, but in Ulk1-knockdown cells LC3-puncta were absent despite rapamycin treatment (Figure S4B bottom panels). Thus, unlike conventional autophagy, the acquisition of LC3 on LdCVs is independent of Ulk1. Treatment with rapamycin has also been described to enhance levels of LC3 associated with pathogens, including *S*. Typhimurium^77^. However, treatment with this drug did not enhance LC3-decoration of *L. dumoffii*. Rather levels were slightly decreased (Figure S4C).

Taken all together, we concluded that LC3-recruitment to a subset of Dot/Icm T4SS^+^ LdCVs is mediated by LAP, a process independent of the mTOR-Ulk1 axis. Recently Rubicon was reported to be involved in LAP induction by fungal infection^78^. In BMDMs obtained from Rubicon^−/−^ knockout mice, LC3-positive LdCVs were formed at a lower level than the Rubicon^+/−^ control (Fig. 5E), providing further support for this notion. Further, unlike other pathogens that gain LC3 through multiple mechanisms, such as *Salmonella*, LAP appears to be the major mechanism targeting *L. dumoffii* for LC3-decoration.

### Membrane damage is not associated with LAP induced by *L. dumoffii* infection

**Figure 6:**
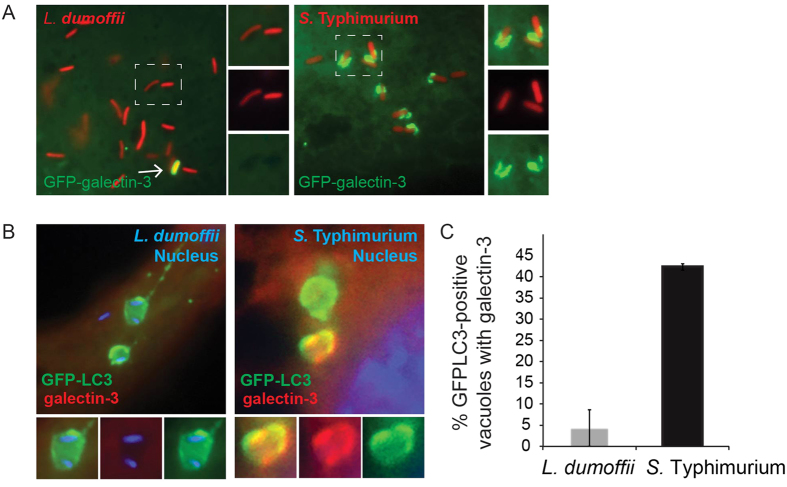
LC3-associated LdCVs preclude galectin-3. (**A**) HeLa cells stably expressing FcγRII receptor were co-transfected with pTLR2 and either GFP-galectin-3. Images shown are at 2 hours post-infection for opsonized *L. dumoffii* (left) and *S*. Typhimurium (right), both organisms harbour plasmids for constitutive expression of mCherry (red). (**B**) Representative fluorescent micrographs showing co-localization of GFP-LC3 and galectin-3 on SCVs but not LdCVs. RAW264.7 cells stably expressing GFP-LC3 were infected with either *S*. Typhimurium (right) or *L. dumoffii* (left). Bacteria are shown in blue (Hoescht 3342). Galectin-3 was detected by indirect immunofluorescence using an anti-galectin-3 antibody (Santa Cruz #sc-23938). (**C**) Quantification of assay shown in panel B. Data are average of three independent experiments, each performed in triplicate.

Classical autophagy is often associated with membrane damage^25^,^79^, but whether membrane damage is important for LAP-activation is not clear. Galectin-3 has been used a marker for damaged phagosomes^80^. Thus, we examined the localization of galectin-3 on LdCVs as a measure of membrane integrity. As a control we again utilized *S*. Typhimurium, as galectin-3 has been found on SCVs^42^. Unlike SCVs, LdCVs were largely devoid of galectin-3 (Fig. 6A). To further examine the relationship between LAP-targeting of LdCVs and membrane damage, we next examined LC3-positive phagosomes for the presence of galectin-3. Whereas LC3-positive SCVs were commonly positive (~43%) for galectin-3, this marker was rare (<5%) on LC3-positive LdCVs (Fig. 6B,C). Taken together, our data demonstrated that LAP-activation on LdCVs is independent of membrane damage.

### LAP induced by *L. dumoffii* infection involves NADPH oxidases and diacylglycerol

**Figure 7:**
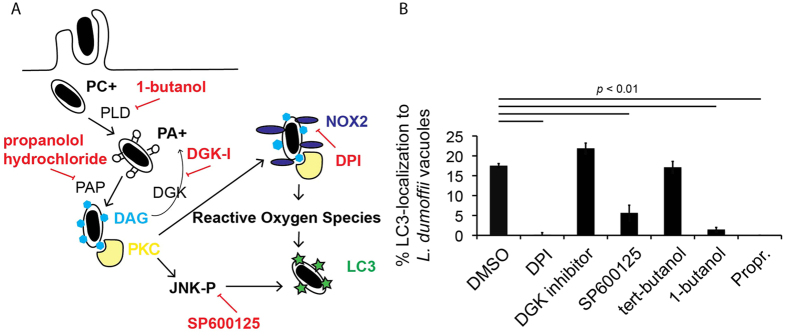
Host factors involved in LAP-targeting of LdCVs. (**A**) Diagram showing proposed pathway of DAG formation on bacterial phagosomes using the host enzymes phospholipase D (PLD) and phosphatidic acid phosphatase (PAP). DAG is recognized by protein kinase C (PKC), which phosphorylates an essential component of NADPH oxidases and c-Jun N-terminal kinase (JNK). Pharmacological inhibitors are shown in red. (**B**) Quantification of single phagosome analysis of LC3-recruitment to *L. dumoffii*-containing vacuoles in THP-1 cells at 3 hours post-infection. Cells were treated with growth media containing DMSO, DPI (10 μM), DGK inhibitor R59 022 (10 μM), SP600125 (20 μM), tert-butanol (negative control), 1-butanol or propranolol hydrochloride (250 μM) at the point of infection and were maintained throughout the experiment. Data are average of three independent experiments, each performed in triplicate. In all relevant experiments, significance was determined using the two-tailed Student’s t-test.

Nicotinamide adenine dinucleotide phosphate (NADPH) oxidases produce reactive oxygen species (ROS) in response to phagocytosis or inflammatory mediators, and this ‘respiratory burst’ acts directly to kill invading organisms^81^. Additionally, a role for the NADPH oxidases in recruitment of LC3 to phagosomes (Fig. 7A), including phagosomes harboring *S*. Typhimurium and *L. monocytogenes*, was demonstrated using the NADPH oxidase inhibitor diphenyleneiodonium (DPI) and Nox2^−/−^ mice^45^,^46^. Similarly, we found addition of 10 μM DPI upon *L. dumoffii* infection drastically impaired LC3-acquisition (Fig. 7B). This suggests that NADPH oxidases are involved in activating LAP upon *L. dumoffii* infection.

In addition to other autophagic recognition mechanisms^38^,^39^,^40^,^41^,^42^,^43^,^82^, both *S*. Typhimurium and *L. monocytogenes* are decorated with diacylglycerol (DAG) during infection and this signal has been shown to be involved in LC3 association^46^,^47^,^83^. Manipulation of DAG levels using chemical inhibitors was found to perturb LC3-association with both organisms. Similarly, we found that specific chemical interventions that perturb DAG production (Fig. 7A), via inhibition of phospholipase D (PLD) by 1-butanol or phosphatidic acid phosphatase (PAP) by propranolol hydrochloride (Propr.), severely reduced association of LC3 with LdCVs (Fig. 7B). In contrast *tert*-butanol, an isomer of 1-butanol that has no inhibitory effect on PLD, had no effect on LC3 association with LdCVs. Normally, DAG kinase (DGK) reduces DAG levels by conversion of DAG to phosphatidic-acid. By inhibiting DGK with DGK inhibitor (R59022), which is expected to promote DAG accumulation, we reproducibly observed slightly but not significant enhanced levels of LC3-association with LdCVs (Fig. 7B). Furthermore, our data suggests both PAP and PLD function during *L. dumoffii* infection in a manner promoting LC3-association. The link between DAG and LC3 is thought to be PKCδ-binding via its C1-domain to vacuolar DAG, and subsequent activation of JNK and NADPH oxidases^47^. Indeed, inhibition of JNK activation using the drug SP600125 also reduced association of LC3 with LdCVs, though not as completely as inhibition of DAG production or NADPH oxidases (Fig. 7B). Taken together, our results support a role for both TLR2 and ROS generation via DAG-NADPH oxidases axis in triggering LAP.

### LAP limits intracellular survival of *L. dumoffii*

**Figure 8:**
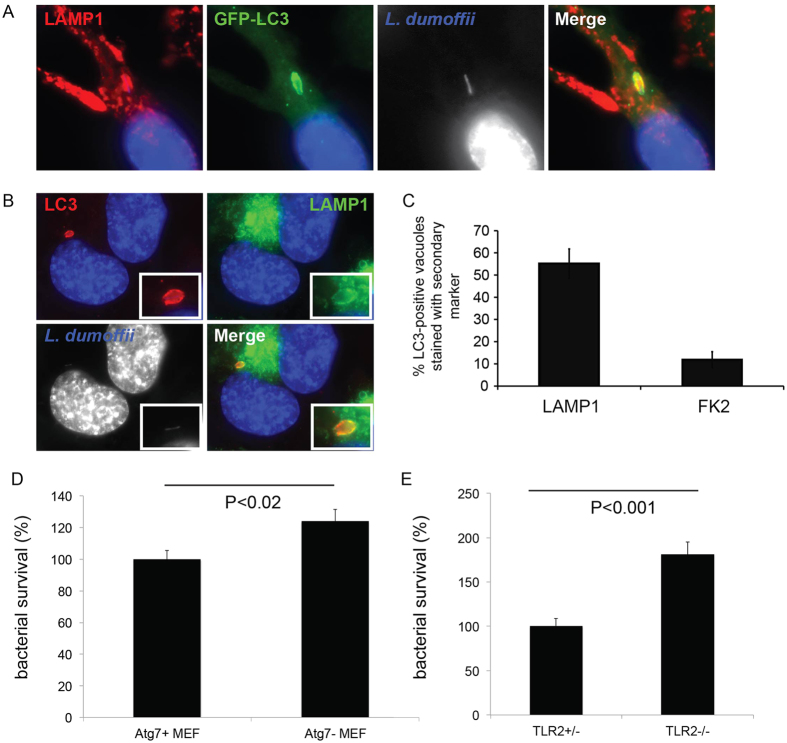
LAP-targeting of LdCVs results in *L. dumoffii* degradation. (**A**) Representative fluorescent image showing co-localization of GFP-LC3 and LAMP1 to an LdCV at 3 hours post-infection in RAW264.7 cells. LAMP1 was detected by indirect immunofluorescence using anti-LAMP1 (clone H4A3; Santa Cruz #sc-20011). Hoescht 33342 (blue) was used to stain both nuclear and bacterial DNA. (**B**) Images show dual antibody labelling of LC3 and LAMP1 on an LdCV at 3 hours post-infection in THP-1 cells. (**C**) Quantification at 3 hours showed 55% of LC3-positive vacuoles also possessed the lysosomal marker LAMP1 in THP-1 cells. However, ubiquitin is not a common feature of the LC3-positive *L. dumoffii* vacuole. (**D**) *Atg7*^+/−^ and *Atg7*^−/−^ knockout MEF cells were infected with *L. dumoffii* ∆*flaA* and intracellular survival was assessed by counting colony forming units at five hours post-infection. (**E**) Bone marrow-derived macrophages from *Tlr2*^+/−^ and *Tlr2*^−/−^ mice were infected with *L. dumoffii* ∆*flaA* and intracellular survival was assessed by counting colony-forming units at five hours post-infection. *P*-values determined by the two-tailed Student’s t-test are shown.

To elucidate whether this host response limits *L. dumoffii* inside host cells, or whether *L. dumoffii* utilizes Atg-proteins for its intracellular survival strategy we first examined whether compartments positive for both LC3 and LAMP-1 exist. Classically, when LC3-positive autophagosomes fuse with lysosomes, autophagolysosome will be decorated with both LC3 and LAMP-1 before degradation of phosphatidylethanolamine (PE)-conjugated LC3 found within the lumen of the membrane-bound compartment. In RAW cells stably expressing GFP-LC3, LC3-positive LdCVs were also positive for LAMP1 by indirect immunofluorescence (Fig. 8A). In THP-1 cells, using dual staining, we found that at 3 hours post-infection ~50% of LC3-positive LdCVs were also positive for LAMP-1 (Fig. 8B,C). Thus, delayed acquisition of LAMP-1 of LdCVs is associated with LAP. As a control we also examined LC3-positive LdCVs for the presence of ubiquitin. LdCVs were rarely positive (~10%) for both LC3 and ubiquitin, consistently with our previous finding that only ~2% of total LdCVs are ubiquitin-positive (Fig. 3B).

To examine the fate of bacteria within LC3-positive LdCVs we then performed live imaging experiments. We did not observe LC3-positive LdCVs being expelled from the cells, as is the case for uropathogenic *E. coli*^67^, but rather the mCherry expressing bacteria within LC3-positive compartments disappeared over time (Figure S5). Given that a number of pathogens interfere with host processes that acidify lysosomes to create a niche in which to replicate, we also confirmed that LdCVs become acidified by live imaging of RAW cells stably expressing GFP-LC3 infected with Hoescht-stained *L. dumoffii* strains in the presence of LysoTracker^®^ Red (Figure S6). As a positive control we tracked ∆*dotA* mutant *L. dumoffii*. ∆*dotA* mutants were found within LC3-negative but acidic (lysotracker-positive) compartment shortly after infection, which is consistent with trafficking through the canonical endocytic pathway (Figure S6A, top panels). Conversely, acidified *L. dumoffii* vacuoles were not observed at one hour post-infection (Figure S6A, bottom panels). However, at 3 hours, LC3-positive but not acidified vacuoles were mainly observed and at 4–5 hours a positive lysotracker signal was associated with ~10% of *L. dumoffii* and LC3-positive LdCVs were rare (Figure S6B). Thus, acidification occurs post acquisition of LC3. This data further supports degradation of pathogenic *L. dumoffii* that are recognized by Atg-components.

Because LAP of *L. dumoffii* results in bacterial degradation, we hypothesized that this innate immune response acts to restrict *L. dumoffii*. We examined this hypothesis using a bacterial viability-based assay. In wild-type MEF cells, ~25% more *L. dumoffii* remained viable in comparison with in Atg7 knockout MEF cells at five hours post infection (Fig. 8D). Likewise, in BMDMs derived from TLR2^−/−^ knockout mice, ~80% more *L. dumoffii* remained viable in comparison with in BMDMs derived from TLR2^+/−^ mice (Fig. 8E). Taken together, these results demonstrated that LAP can limit intracellular survival of a virulent bacterial pathogen that resides within a membrane-bound compartment within host cells.

### RavZ is sufficient to block LAP induced by *L. dumoffii* infection

**Figure 9:**
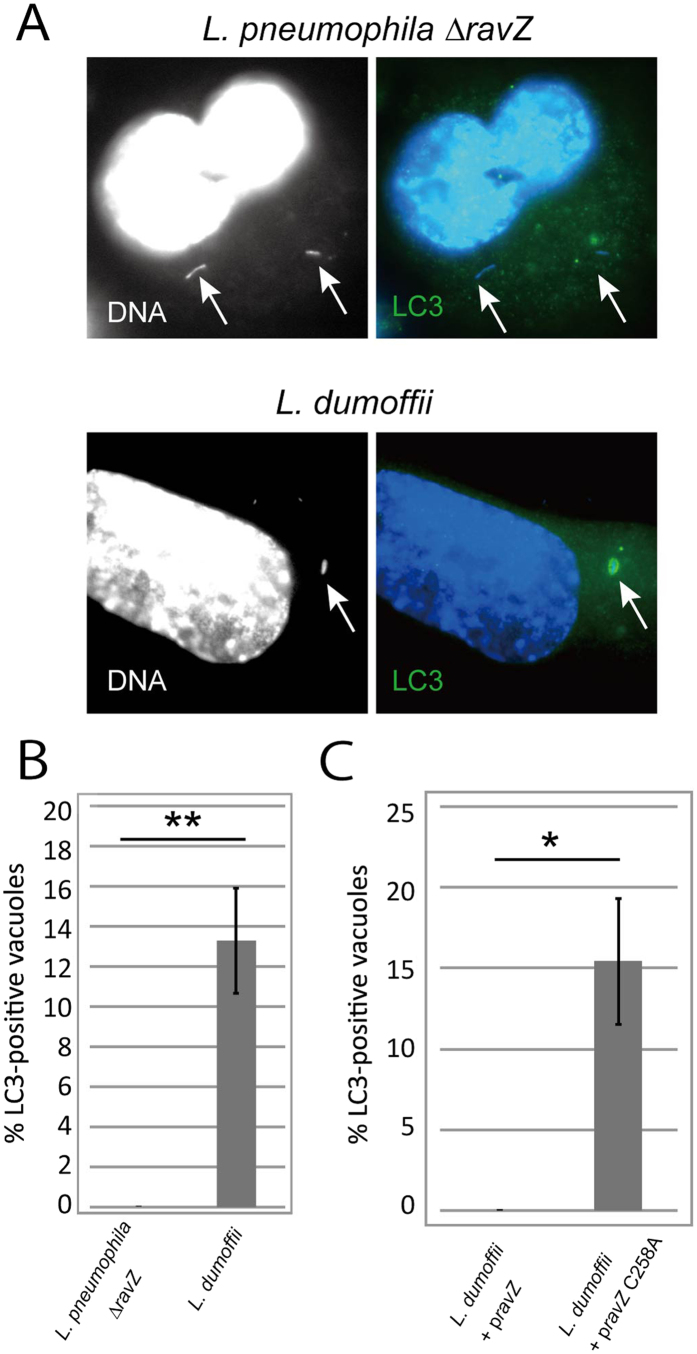
Effects of RavZ in LC3-recruitment to LpCVs and LdCVs. (**A**) THP-1 cells were infected with a *L. pneumophila* strain lacking *ravZ* or a wild-type *L. dumoffii* which does not encode *ravZ* for 3 hours. LC3 was stained with LC3 antibodies. Bacteria were visualized with DAPI-staining (white arrows). (**B**) Quantification of LC3-association on Δ*ravZ* LpCVs and LdCVs. (**C**) THP-1 cells were infected with a wild-type *L. dumoffii* having a pMMB207-derived plasmid expressing *L. pneumophila ravZ* or its catalytic mutant *ravZ*C258A for 3 hours. LC3-associated LdCVs were quantified as in (**B**). Significance of the results was determined using the two-tailed Student’s t-test; **p < 0.01, *p < 0.03.

Some *L. pneumophila* strains including Lp01 we used in this study encode the effector protein RavZ, which irreversibly deconjugates LC3 from phospholipid membrane^17^ and thus interferes LAP^78^. To explore the exact role of RavZ in LAP avoidance of LpCVs, we examined ubiquitin and LC3 recruitment to LpCVs containing a RavZ detetion strain in THP-1 cells at three hours post infection, In this condition we observed LC3 recruitment to LdCVs but rarely to LpCVs (Fig. 9A,B) like LpCVs containing the wild-type strain (Fig. 3B). This clearly indicates that the LAP avoidance of *L. pneumophila* is not solely due to the effector RavZ function.

On the other hand, *L. dumoffii* NY23 used in this study does not encode RavZ ortholog. When *L. pneumophila* RavZ or its catalytic mutant derivative (RavZC258A) was ectopically expressed in *L. dumoffii*, the resulting LdCVs failed to acquire LC3 in a RavZ activity-dependent manner (Fig. 9C), suggesting that the catalytic activity of RavZ is sufficient to block LAP as previously reported^78^.

## Discussion

**Figure 10:**
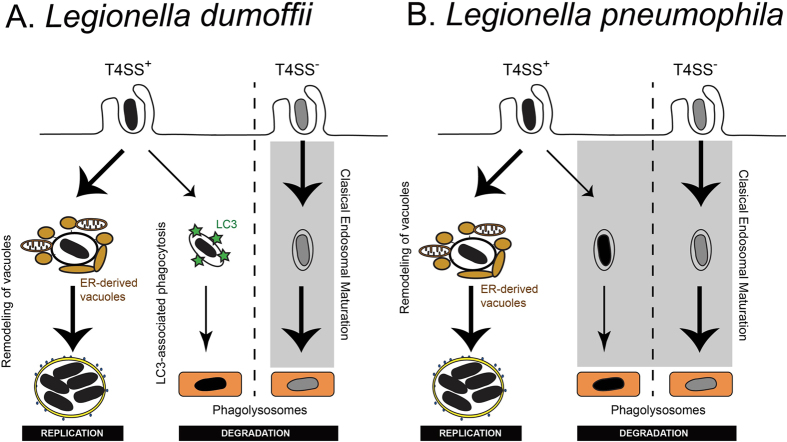
Proposed three alternative fates of intracellular *Legionella*. *L. dumoffii* lacking ability to translocate effectors (without Dot/Icm T4SS) are cleared through endocytic maturation and trafficking to degradative lysosomes (shaded). However, the fate of virulent (with Dot/Icm T4SS) *L. dumoffii* is more complex. Though the majority (~80%) will subvert canonical endocytic maturation and go on to replicate inside ER-like compartments, some (~20%) will be degraded following initiation of LC3-associated phagocytosis (LAP). Thus, the nature and kinetics of unproductive *L. dumoffii* trafficking differ substantially from those of *L. pneumophila*.

To lay a foundation for studies examining species-specific *Legionella* effector functions during intracellular infection, we set out to phenotypically compare *L. pneumophila* and the largely unstudied pathogen *L. dumoffii. L. dumoffii* was found to share some of *L. pneumophila*’s intracellular survival strategies, namely inhibition of the canonical endocytic pathway and creation of an ER-like intracellular niche. However, we identified two points of difference between the organisms: *L. dumoffii*’s decoration with LC3 and lack of vacuole ubiquitination. Extending this insight, we show that LAP is the main process for LC3-decoration of *L. dumoffii* in host cells. LC3-decoration of LdCVs was also found to be Dot/Icm T4SS-dependent (Fig. 2). Thus, we propose that the majority of pathogenic (T4SS^+^) *L. dumoffii* survive in host cells by creating an ER-like intracellular niche, but a portion of *L. dumoffii* are recognized by the host and decorated with LC3 in a ubiquitin-independent manner consistent with LAP (Fig. 10). Our data are consistent with the previous report that found ~20% of intracellular *L. dumoffii* were LAMP-1 positive at 4 hours post-infection in J774 mouse macrophages^84^.

The relationship between Dot/Icm-dependent LdCV maturation and LAP prompts us to question whether there is a link between phagosome maturation and LAP initiation. Regarding the balance of LAP-targeting over autophagy, our data indicates that vacuolar integrity is a likely contributing factor. Based on the lack of galectin-3 (or any other galectins we tested) association with LdCVs (Fig. 6), LAP-activation appears independent of membrane damage. Thus, vacuolar integrity of LdCVs may skews recognition away from membrane-damage responsive autophagy^25^,^79^. Alternatively, it remains possible that our membrane damage marker (galectin-3) is somehow removed from LdCVs. LAP’s predominance may simply be due to the absence of phagosomal ubiquitin. In this scenario, TLR- and DAG-signaling and delayed phagosome maturation would provide positive LAP-activation signals and ubiquitin-negative intact phagosomes would avoid activation of classical autophagy.

Why LdCVs are subjected to LAP but LpCVs are not is an intriguing question. The *L. pneumophila* strain Lp01 used in this study produces the effector protein RavZ, which has an activity to irreversibly deconjugate LC3 and thus inhibit autophagy-related pathways including macroautophagy and LAP^17^,^78^. We examined the behavior of a RavZ deletion derivative and found that RavZ is not required for the LAP avoidance by *L. pneumophila* (Fig. 9A,B). This suggests that either *L. pneumophila* has additional effectors to inhibit LAP or *L. dumoffii* has an effector to promote LAP. The effector Spl^36^ is one of such candidates which may inhibit LAP, however, *L. dumoffii* appears to encode the Spl ortholog. On the other hand, *L. dumoffii* ectopically producing RavZ failed to engage in LAP (Fig. 9C), indicating that *L. dumoffii* lacks sophistication to have a single effector protein like RavZ which efficiently blocks autophagy-related pathways, unlike some strains of *L. pneumophila*. Clarification of molecular mechanisms underlying *L. dumoffii* engagement in LAP awaits future study.

A remaining question is the physiological importance of LAP for bacterial restriction in the context of other immune defense mechanisms. Recently, LAP has been proposed to facilitate enhanced antigen presentation by delaying phagosome maturation^85^. It is tempting to speculate that in higher eukaryotes, LAP-mediated enhanced antigen presentation by macrophages infected with *L. dumoffii* engages innate or adaptive responses that limit disease progression, whereas the LAP-avoiding pathogen like *L. pneumophila* avoids this enhanced host response and causes more disease.

Rubicon is a Class III PI(3) kinase-associated protein^86^, which recruits PI(3) kinase to phagosomes and supports prolonged localization of PI3P^78^. Then Rubicon interacts with the NOX2 complex recruited in a PI3P-mediated manner and stabilizes the NOX2 complex, resulting induction of ROS production^78^. In contrast, LpCVs were reported to acquire PI3P right after infection in a model natural host *Dictyostelium discoideum*^87^, but soon PI4P becomes a dominant phosphoinositide species, which possibly involves the concerted actions of host PI(4) kinases, and host and bacterial phosphoinositide phosphatases^88^,^89^,^90^,^91^. Currently phosphoinositide dynamics on LdCVs are not known, and future studies focusing on PI3P pool on LAP-targeted versus LAP-escaping LdCVs will shed light on the requirement in LAP induced by fungal and bacterial infection, and the difference in fates of LdCVs and LpCVs. Furthermore LAP-targeting of LdCVs may be used as a model system examining the finer mechanistic details of LAP, particularly induced by bacterial infection.

## Materials and Methods

### Reagents

Unless otherwise noted, all chemicals were purchased from Sigma. Rapamycin was purchased from Santa Cruz (sc-3504). Restriction and molecular cloning enzymes were purchased from New England Biolabs or Toyobo Co. Ltd. Primary antibodies used include rabbit polyclonal antibodies to *L. pneumophila* (Biodesign #B65051G) and *Legionella dumoffii* (Denka Seiken). Primary antibodies used for immunofluorescence experiments are listed in figure legends. Secondary conjugate antibodies Alexa Fluor**^®^** 488 (Life Technologies #A11029), Alexa Fluor**^®^** 488 (Life Technologies #A11034), Rhodamine Red-X (Life Technologies #R6393), and Rhodamine Red-X (Life Technologies #R6394) and Alexa Fluor**^®^** 568 (Life Technologies #A11077) were purchased from Thermo Fischer Scientific.

### Bacterial Strains

Bacterial strains used in this study are described in Table S1. *Legionella* strains were grown on charcoal-yeast extract plates or in aces-buffered yeast extract broth as already described^92^. When required, drugs were included in the bacteriological media at the following concentrations: for strains of *Legionella* species, streptomycin 100 μg/ml, chloramphenicol 3 μg/ml and kanamycin 10 μg/ml; for strains of *E. coli*, ampicillin 100 μg/ml, kanamycin 25 μg/ml and chloramphenicol 20 μg/ml; for *Salmonella enterica* serovar Typhimurium (*S*. Typhimurium), chloramphenicol 20 μg/ml.

### DNA manipulations

Bacterial plasmids used in this study are listed in Table S2. pMMB207mCherry was created by cloning mCherry into pAM239. To induce constitutive expression of mCherry an internal region of the lacI repressor was disrupted. To create the ∆*dotA*, ∆*flaA L. dumoffii* and ∆*ravZ L. pneumophila* mutants, the plasmids pSR47SLd∆*dotA*, pSR47SLd∆*flaA and* pSR47SLd∆*ravZ* were created using primer pairs list in Table S3. Clones were sequenced using the primers pSR47Sac1 and pSR47SXba1. The *ravZ* C258A mutation was introduced using QuickChange II Site-Directed Mutagenesis Kit (Agilent Technologies) according to the manufacture’s instruction. The lentiviral vector for the stable expression of GFP-LC3 was generated as follows. The genes encoding GFP and LC3 were amplified using the primer pairs listed in Table S3 with pEGFP-LC3^93^ as a template. The PCR product was subsequently amplified with attB adaptor primers and cloned into pDONR201 (Invitrogen) with Gateway BP Clonase II Enzyme Mix (Invitrogen). The sequence encoding GFP-LC3 was then transferred to the lentiviral expression vector pLEXEF.pur^94^, using Gateway LR Clonase II Enzyme Mix (Invitrogen), to obtain pLEXEF.pur.GFP-LC3.

### Genetic manipulations of bacteria

Strain Ld00 is a spontaneous streptomycin-resistant mutant of *L. dumoffii* NY23. Chromosomal deletion mutants of *L. dumoffii* were made using the pSR47S-based plasmids described above and following the method previously described for *L. pneumophila*^95^. *Legionella* strains constitutively expressing mCherry were created by introduction of pMMB207::mCherry by electroporation using the conditions described for *L. pneumophila*^2^. *S*. Typhimurium expressing pMMB207::mCherry was also created by electroporation and selection on chloramphenicol resistance.

### Tissue culture and media

HEK293 cells stably expressing the FcγRII receptor were obtained from the laboratory of Craig Roy (Yale University, USA)^96^. HeLa cells lines stably expressing the FcγRII were created and kindly provided by Dr. Kohei Arasaki (Tokyo University of Pharmacy and Life Sciences, Japan). Wild-type MEF cells were provided by Dr. Miwa Sasai (Osaka University, Japan). Immortalized Atg7-deficient MEFs were kindly provided by Dr. Tatsuya Saitoh (Osaka University, Japan) on behalf of Dr. Masaaki Komatsu (Niigata University, Japan). TLR2^97^, TLR4^98^, Rubicon^99^ KO mice were previously described. Murine BMDMs were obtained from mice as previously described^100^, and used fresh or stored as described^101^. All mammalian cell lines were maintained in 5% CO_2_ at 37 °C. Mouse embryonic fibroblasts (MEF), HeLa, HEK293 and FcγRII-expressing stable variants^96^ were maintained in Dulbecco’s modified Eagle’s growth medium (Gibco, Life Technologies) supplemented with 10% fetal calf serum. THP-1 cells were routinely passaged as non-adherent cells. RAW264.7 and THP-1 cells were cultured in RPMI 1640 medium (Gibco, Life Technologies) supplemented with 10% fetal calf serum. For knockdown experiments, HEK293 cells were seeded into 24-well tissue culture plates to achieve 40% confluency 24 hours later when plasmid DNA and siRNA was added to wells. Two-days later, media was changed to DMEM plus 5% FBS for the final overnight incubation. Infection experiments in complete media were performed at 72 hours post-transfection. For infection experiments with THP-1 cells, cells were differentiated in tissue culture wells at densities of 2 × 10^5^ cells per well of the 24-well dishes and 5 × 10^4^ cells for the 96-well dishes. Three days prior to use, THP-1 cells were incubated in media containing phorbol 12-myristate 13-acetate (PMA, Sigma). After 24–48 hours media was replaced with fresh media lacking PMA. The next day, infection experiments were performed. To create the RAW 264.7 cell line stably expressing GFP-LC3, the ViraPower Lentiviral Expression System (Invitrogen) was used to produce lentiviruses, and the resulting viral supernatant was used to transduce the lentiviral construct into RAW 264.7 cells. Subsequently, stable GFP-LC3-expressing cells were established by selection with puromycin (Clontech). RAW cells stably expressing GFP-LC3 were cultured in RPMI 1640 medium supplemented with 10% fetal calf serum.

### Host cell manipulations

HEK293 and HeLa cell transfections were performed using Lipofectamine 2000 (Invitrogen) or Lipofectamine LTX+ (Invitrogen) according to the manufacturer’s instructions. For co-transfection assays in HEK293 cells, plasmid DNA (200 ng) was mixed with siRNA (50 nM) and Lipofectamine 2000 before co-transfection into cells. Scrambled siRNA and Ulk1 siRNA for RNAi experiments were ordered from Sigma Genosys. The sequence of the Ulk1 siRNA used was previously reported^102^. Lipofectamine LTX+ (Invitrogen) was used to transfect MEF cells using 1.75 μl of LTX, 0.5 μl ‘plus’ reagent and 0.5 μg DNA per well of the 24-well dishes.

### Bacterial infections

Infections with *L. pneumophila* or *L. dumoffii* were performed with bacteria growth in 2-ml AYE both cultures for 20 hours. The final OD_600_ of *L. dumoffii* cultures used for infection was ~2.8 (range of 2.7 to 3.2), whereas *L. pneumophila* cultures were used at ~4.0 (range of 3.8 to 4.4). HEK293 FcγRII and HeLa FcγRII cells were infected with *L. dumoffii* at a multiplicity of infection (moi) of one as previously described for *L. pneumophila*^90^. For non-FcγRII-mediated uptake, an moi of 100 to 300 was used. For infection of RAW264.7 cells stably expressing GFP-LC3, THP-1 and MEF cells an moi of 10 to 30 was used. For dual infection experiments of *L. pneumophila* mCherry and *L. dumoffii*, 10 times more *L. pneumophila* was used than *L. dumoffii* because we observed better uptake of *L. dumoffii* than *L. pneumophila*. To propagate *Salmonella* for infections, overnight cultures were diluted to OD_600_ of 0.17 and grown for ~3 hours until the OD_600_ reached 1.8–2.5, and finally bacteria were added to cells at an moi of 100. At 20 min post-infection, 100 μg/ml gentamycin was added and maintained until cells were washed and fixed. In all experiments cells were fixed with 4% PFA for 15 min at 37 °C. After staining coverslips were stained with Hoechst at 1 μg/ml and mounted using the ProLong gold antifade reagent (Invitrogen). For growth curves in mammalian cells an MOI of 0.5 was used and performed as previously described^90^. For experiments using C57BL/6 BMDM and MEF cells, infections were performed using *L. dumoffii*∆*flaA*.

### RT-PCR

Knockdown efficiency was validated by quantitative RT-PCR using Thunderbird^TM^ SYBR**^®^** qPCR mix on cDNA synthesized by the SuperScript^TM^ III (Invitrogen#18080–051) kit from total RNA extracted from cells at 72 h post-knockdown using the RNeasy kit (Qiagen). The primer pairs used to assess transcript levels are listed in Table S2.

### Microscopy

Epifluorescence micrographs were taken using a TE2000 (Nikon) inverted microscope and this microscope was used for all counting experiments. Confocal micrographs were taken using a LSM510 microscope (Zeiss) with a 100x/1.4 numerical aperture objective, or a Fluoview FV10i (Olympus) microscope with a 60×/1.35 numerical aperture objective. Correlative light electron microscopy (CLEM) experiments were performed as previously described^103^. Basically, RAW264.7 cells stably expressing EYGP-LC3 were cultured on glass bottom dishes (with grids) and then infected with mCherry-expressing *Legionella dumoffii* for 2.5–3 hours. After fixing, confocal images were taken before processing the samples for electron microscopy.

### Survival assays in MEFs

To perform the *L. dumoffii* survial assay in Atg7+ and Atg7- MEF cells, MEFs were seeded at 5 × 10^4^ per well in 24-well format - to reach ~80% confluency at the time of infection. Fresh 2-day heavy patch *L. dumoffii∆flaA* expressing mCherry were grown overnight in AYE plus chloramphenicol broth until OD_600_ reached 2.8–3.0. Bacteria were added to wells at 2 × 10^5^ per well. Plates were spun at 1000 rpm for 5 minutes and then incubated for 5 hours under standard cell culture conditions. At 5 hours post-infection, wells were gently washed two times with PBS to remove extracellular bacteria. Cells were then lysed in 1 ml of sterile water per well. To promote host cells lysis, cells were freezed and thawed before vigorously resuspending the contents of each well and plating for bacterial viability on CYE media. For each cell type, sixteen independent wells were assessed from four separate plates and each well was plated in duplicate. Data are shown compared to control cells (wild-type) average bacterial survival, which was normalized to 100%. Significance was determined by the students’ T-Test.

### Survival Assays in Macrophages

Differentiated BMDMs were seeded in 24-well plates at 1.5 × 10^5^ cells per well. The following day, *L. dumoffii∆flaA* expressing mCherry were added at 5 × 10^5^ per well and spun for 5 min at 1000 rpm. At 5 hours post-infection, wells were treated as described for MEF cells with a final well volume of 1 ml. One hundred μl of 1/100 dilutions were plated to enumerate bacterial survival. In triplicate experiments, six wells were assessed for each cell type. Data are shown compared to control cells (wild-type) average bacterial survival, which was normalized to 100%. Significance was determined by the students’ T-Test.

### Statistical Analysis

To statistically assess significance, calculations were performed using the paired Student’s t-test (homoscedastic two-tailed, paired) using Excel software (Microsoft). In all graphs error bars represent standard error of the mean (SEM). Where appropriate, p values or ns for not significant were denoted.

### Ethics statement

All animal experiments were performed in accordance with the institutional guidelines and were approved by the Animal Care and Use Committee of the Research Institute for Microbial Diseases, Osaka University, Japan (Biken-AP-H26-10-0, IFReC-AP-H27-07-0).

## Additional Information

**How to cite this article:** Hubber, A. *et al*. Bacterial secretion system skews the fate of *Legionella*-containing vacuoles towards LC3-associated phagocytosis. *Sci. Rep.* **7**, 44795; doi: 10.1038/srep44795 (2017).

**Publisher's note:** Springer Nature remains neutral with regard to jurisdictional claims in published maps and institutional affiliations.

## Supplementary Material

Supplementary Information
