# Supplementary material for: Bacterial secretion system skews the fate of Legionella-containing vacuoles towards LC3-associated phagocytosis
Source: Sci Rep. 2017 Mar 20;7:44795. doi: 10.1038/srep44795 (PMC5357938; doi:10.1038/srep44795)
Supplement: Supplementary Information [file srep44795-s1.pdf]

Supplemental Information to

Bacterial secretion system skews the fate of *Legionella*-containing vacuoles towards LC3-associated phagocytosis.

Andree Hubber[1], Tomoko Kubori[1], Cevayir Coban[2], Takeshi Matsuzawa[3], Michinaga Ogawa[4], Tsuyoshi Kawabata[5], Tamotsu Yoshimori[5] and Hiroki Nagai[1*]

1. Research Institute for Microbial Diseases, Osaka University, 3-1 Yamadaoka, Suita, Osaka 565-0871, Japan

2. Laboratory of Malaria Immunology, Immunology Frontier Research Centre, Osaka University, 3-1 Yamadaoka, Suita, Osaka 565-0871, Japan

3. Graduate School of Life and Environmental Sciences, Osaka Prefecture University, Rinku Ohrai Kita 1-58, Izumisano, Osaka 598-8531, Japan

4. Department of Bacteriology I, National Institute of Infectious Diseases, Toyama 1-23-1, Shinjuku-ku, Tokyo 162-8640, Japan

5. Department of Genetics, Osaka University Graduate School of Medicine, 2-2 Yamadaoka, Suita, Osaka 565-0871, Japan

*To whom correspondence should be addressed

Email: hnagai@biken.osaka-u.ac.jp

Telephone: +81 6 6879 8361

Fax: +81 6 6879 8361

Figure S1. The *L. dumoffii* replicates in ER-like vacuoles in a Dot/Icm type IV secretion system dependent manner.

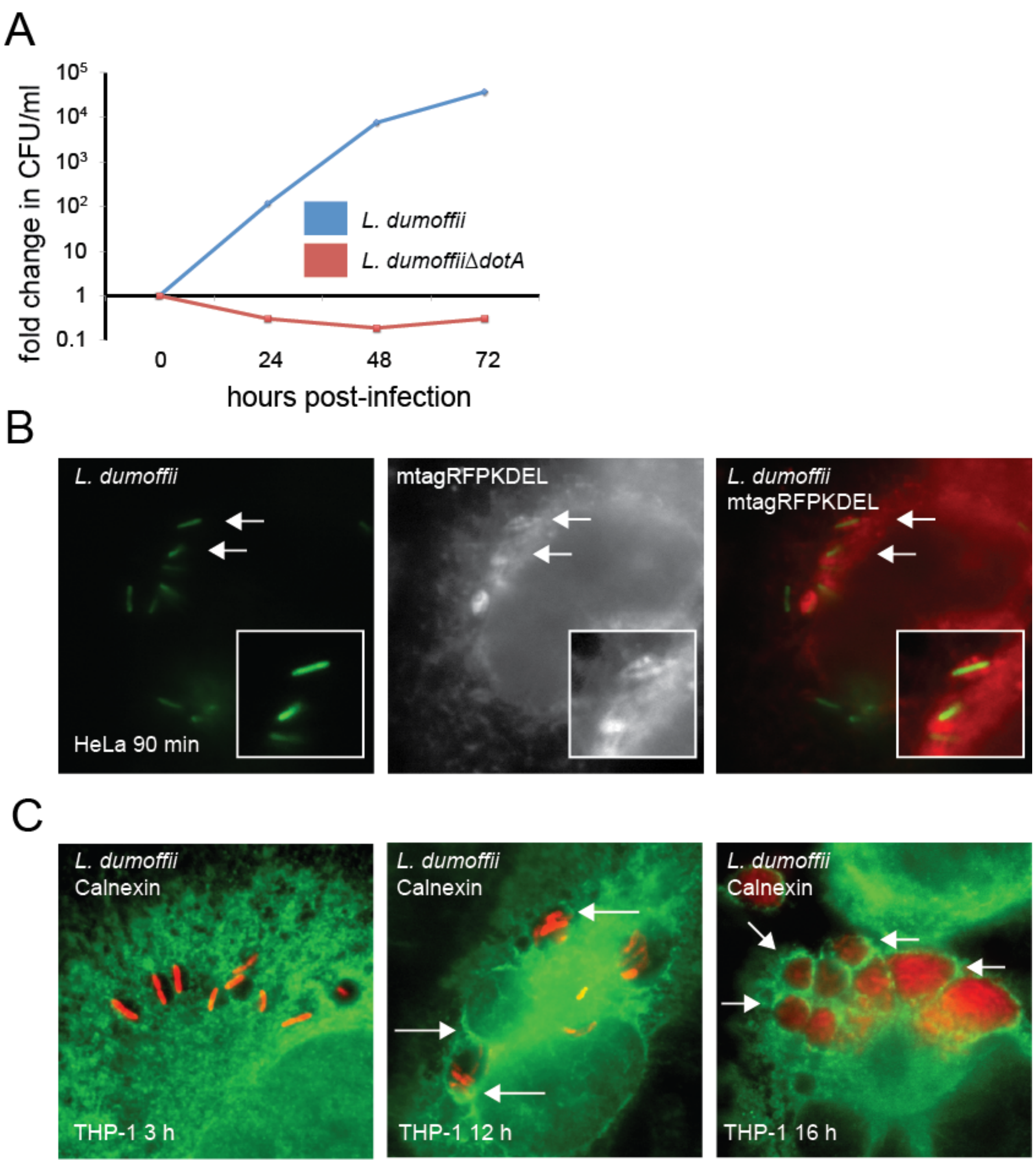

(A) Graph showing fold change in CFU/ml of *L. dumoffii* NY23 Str^{R} and *L. dumoffii*Δ*dotA* NY23 Str^{R} in THP-1 cells over 3 days. Data are from a single experiment and values shown are the average of three independent infections each performed with triplicate wells. Error bars are ± SEM. (B) Fluorescent image of a HeLa FcγRII cell transfected with mtagRFP-KDEL (red) and infected with *L. dumoffii* constitutively expressing GFPmut3 (green). Clear localization

of the tagged-KDEL to the bacterial phagosome was observed at 90 min post-infection as indicated by white arrows and enlarged in inset. (C) Fluorescent image of human THP-1 cells infected with *L. dumoffii* constitutively expressing mCherry (red). ER-association was assessed by indirect immunofluorescence using a primary antibody raised against the C-terminal (ER luminal) region of calnexin (Enzo#ADI-SPA-860). Image taken at 12 and 16 hours post-infection show positive association of calnexin with *L. dumoffii*-containing phagosomes as shown by white arrows.

Figure S2. Galectin-8 and adapters commonly involved in LC3-recruitment are not associated with LdCVs.

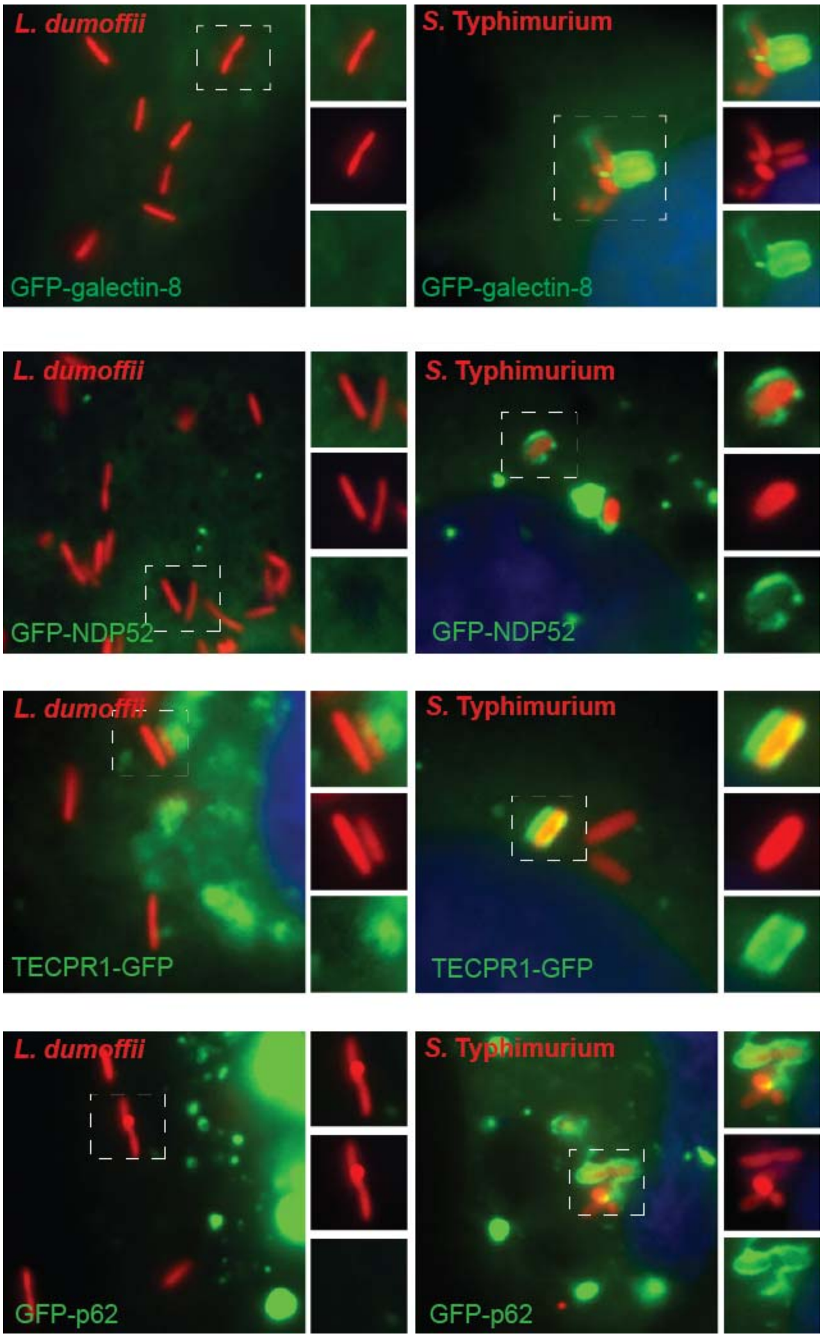

HeLa cells stably expressing FcγRII receptor were co-transfected with pTLR2 (see Figure 6) and a single GFP-expression construct, as shown in fluorescent micrographs; top to bottom: GFP-galectin-8, GFP-NDP52, Tecpr1-GFP, and GFP-p62. Images shown are at 2 hours post-infection for opsonized *L. dumoffii*

(left) and *S.* Typhimurium (right), both organisms harbour plasmids for constitutive expression of mCherry (red).

Figure S3. ***L. dumoffii*∆*flaA*** **replicates robustly in C57BL/6J (B6)-derived macrophages.**

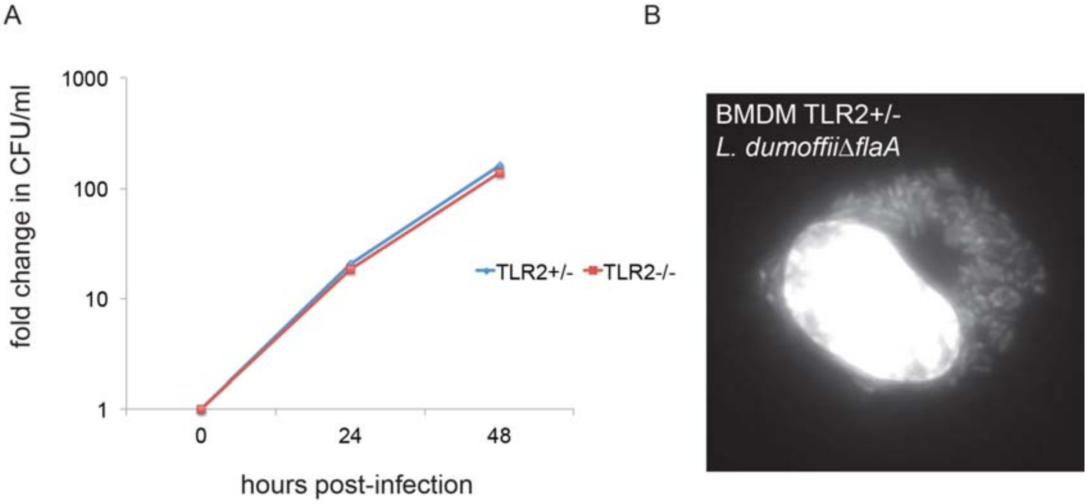

(A). Graph showing fold change in CFU/ml of *L. dumoffii* NY23 ∆*flaA* over 2 days in bone marrow-derived macrophages from TLR2$^{+/-}$ and TLR2$^{-/-}$ C57BL/6J (B6) mice. Data are average of three independent experiments, each performed in triplicate. (B) Representative micrograph of TLR2$^{+/-}$ macrophage infected with *L. dumoffii*∆*flaA.* Nuclear and bacterial DNA is stained with Hoechst 33342.

Figure S4. LC3-recruitment to LdCVs is independent of Ulk1.

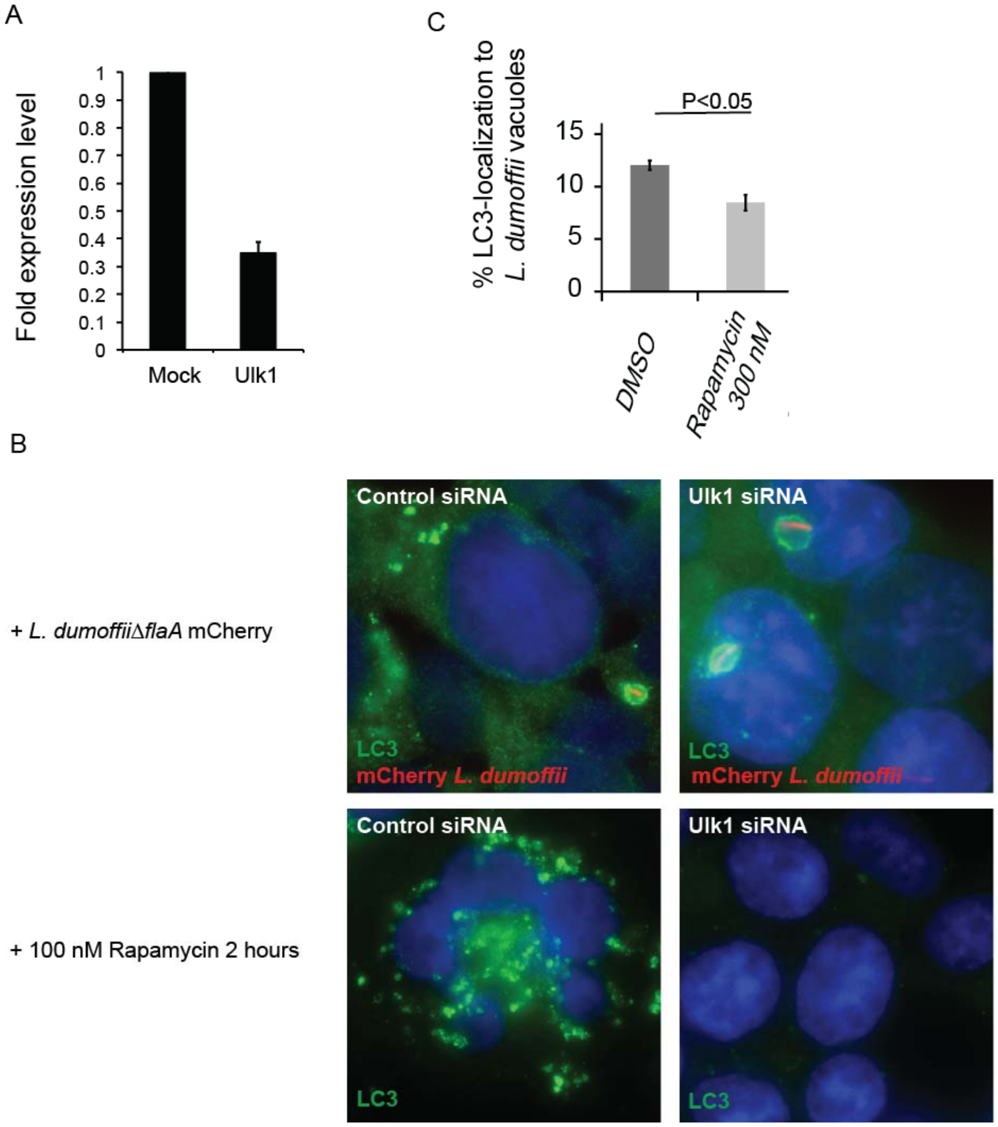

(A) qRT-PCR measuring Ulk1 mRNA levels in HEK293 cells 3 days post-transfection for scrambled (mock) and Ulk1 siRNA-treated cells. Data are normalized to GAPDH and shown as fold expression level compared to mock-transfected cells. (B) Representative images of LC3-localization in HEK293 cells transfected with scrambled (control) or Ulk1-siRNA and infected with mCherry-expressing *L. dumoffii* for 3 hours or treated with 100 nM of the autophagy inducer rapamycin for 2 hours. (C) Quantification of LC3 recruitment to *L. dumoffii* vacuoles at 3 hours post-infection in THP-1 cells treated with

DMSO (control) or Rapamycin. Data is the average of two independent experiments performed in triplicate. Student's t-test was used to determine significance.

Figure S5. Live imaging shows degradation of an LC3-positive *L. dumoffii*.

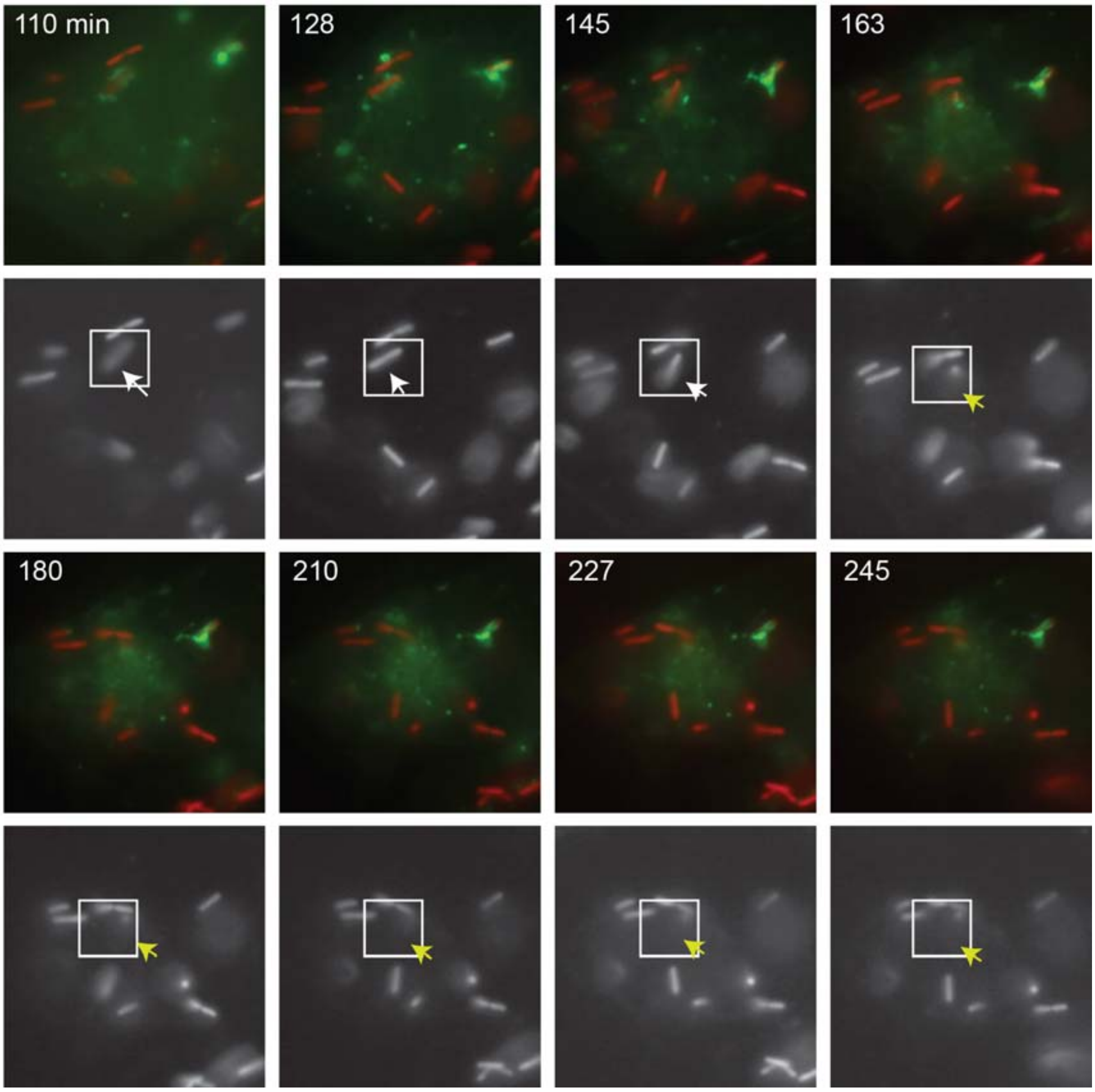

Micrographs show a time course of still images from 110 min to 245 min post-infection for RAW264.7 GFP-LC3 cells infected with mCherry-expressing *L. dumoffii*. Cells were incubated at 37˚C on a heated stage and manually refocused during the course of infection. The boxed bacterium is LC3-positive at 110, 128, and 145 min post-infection (shown by white arrows). However, this bacterium is no longer visible >163 min post-infection (as shown by yellow arrows). In contrast, adjacent bacteria are still visible.

Figure S6. Live imaging shows acidification of a subset of *L. dumoffii* vacuoles at time-points 3-5 hours post-infection, which is temporally consistent with degradation of LC3-positive vacuoles.

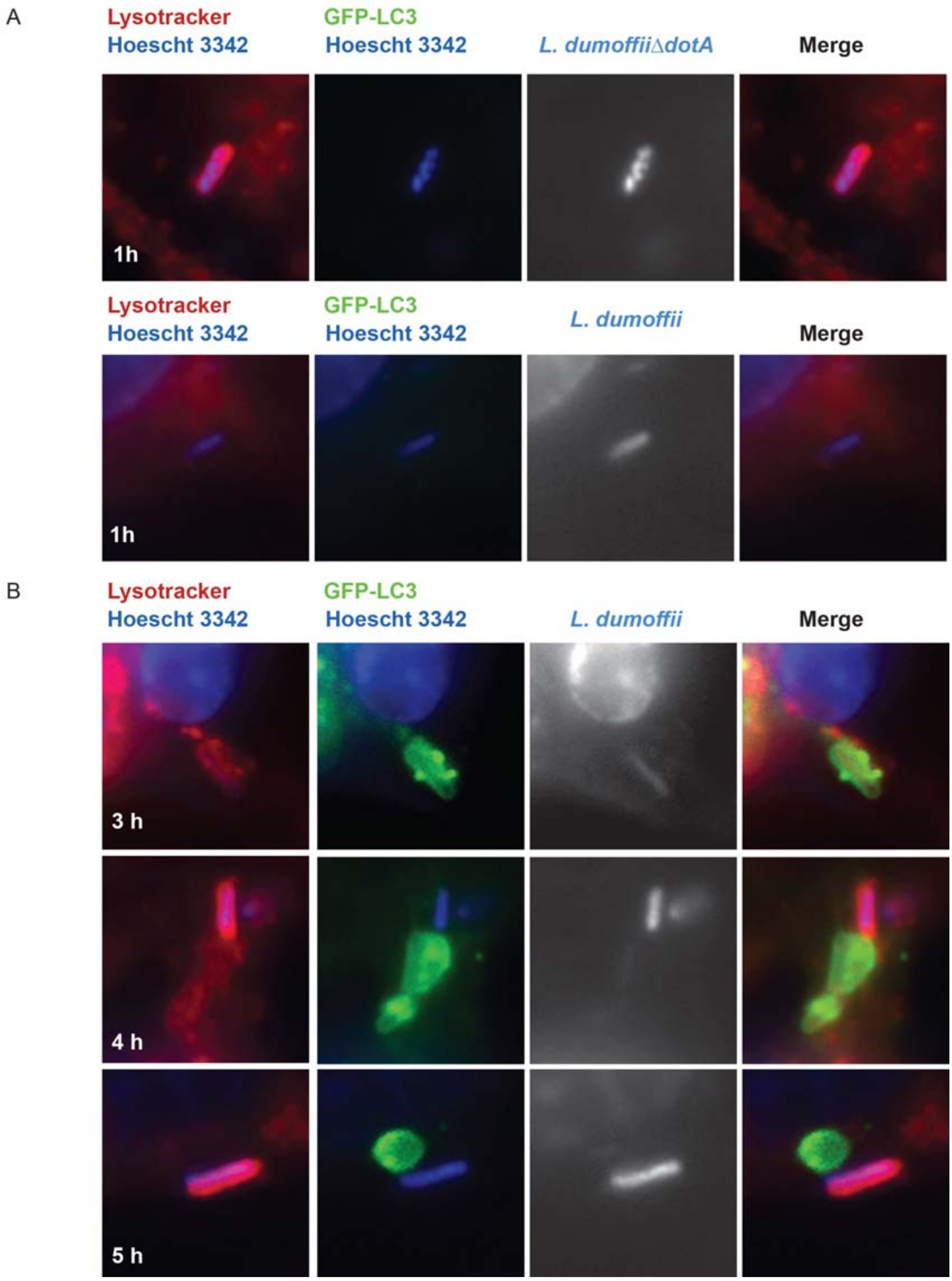

(A) Micrographs show representative images of RAW264.7 GFPLC3 cells infected with Hoescht 3342-stained *L. dumoffii*Δ*dotA* (top) or *L. dumoffii*

(bottom) at 1 hour post-infection. Cells were incubated with 75 nM LysoTracker® Red DND-99 (Life Technologies#L7528) for 1 hour prior to infection and maintained in media throughout assay. Accumulation of the probe in compartments with a low pH is shown by a red signal. (B) Micrographs show representative images of RAW264.7 GFPLC3 cells infected with Hoescht 3342-stained *L. dumoffii* (bottom) at 3, 4 and 5 hours post-infection. Co-localization of LC3 and lysotracker signals was not readily observed. However, the appearance of lysotracker positive signals associating with *L. dumoffii* was more frequently observed at >3 hours post-infection, after the peak of LC3 association.

Text S1. Supplementary Tables S1-S3.

Tables S1-S3 list the bacterial strains, plasmids and primers used in this study, respectively.

Table S1. Bacterial strains

Strain	Genotype (name in study)	Reference
Legionella pneumophila		
Lp01	*Legionella pneumophila* serogroup 1, Lp01 *rpsL*	[1]
Lp01Δ*dotA*	Lp01Δ*dotA rpsL*	[2]
Lp01Δ*ravZ*	Lp01Δ*ravZ rpsL*	This study
Lp01 pmCherry	*Legionella pneumophila* serogroup 1, Lp01 *rpsL* + pMMB207::mCherry	This study
Lp01Δ*dotA* pmCherry	Lp01Δ*dotA rpsL* + pMMB207::mCherry,	This study
Legionella dumoffii		
Ld00	*Legionella dumoffii* NY23 (ATCC33279)	[3]
Ld01	Ld00, Str^R	This study
Ld01 Δ*dotA*	*L dumoffii* Δ*dotA,* Str^R	This study
Ld01 Δ*flaA*	*L dumoffii* Δ*flaA,* Str^R	This study
Ld01 Δ*dotA* pmCherry	*L dumoffii* Δ*dotA* + pMMB207::mCherry Str^R,	This study
Ld01 Δ*flaA* pmCherry	*L dumoffii*Δ*flaA* + pMMB207::mCherry Str^R,	This study

Salmonella enterica serover Typhimurium		
SR-11 χ3181	Prototoroph, mouse-passaged χ3041	[4]
SR-11 pmCherry	SR-11 + pMMB207::mCherry	This study
E.coli		
DH5α	endA1 hsdR17 supE44 thi-1 recA1 gyrA relA1 Δ(lacIZYA-argF)U169, deoR (φ80dlacΔ(lacZ)M15)	Invitrogen
DH5α (λpir)	DH5α λpir phage lysogen	[2]
MT607 pRK600	MT607 *E. coli* containing plasmid pRK600; ColE1 replicon with RK2 transfer genes	[5]

Table S2. Plasmids used.

Plasmid Name	Provided by	Reference
pEGFP-LC3	M. Ogawa	[6]
pLEXEF.pur.GFP-LC3	Kenji Matsuzawa	This study
pEGFP-galectin-3	M. Ogawa	[7]
pEGFP-galectin-8	M. Ogawa	This study
pTecpr1-EGFP	M. Ogawa	[7]
pEGFP-p62	M. Ogawa	[8]
pEGFP-NDP52	M. Ogawa	This study
pTLR2	K. Kobiyama/F. Takeshita	[9]
pTLR4	K. Kobiyama/F. Takeshita	
pSR47S	R6K suicide vector, Km^R, sacB	[10]
pSR47SLdΔ*flaA*	pSR47S containing 5' and 3' flanking genomic regions of *L. dumoffii flaA* (for gene deletion)	This study
pSR47SLdΔ*dotA*	pSR47S containing 5' and 3' flanking genomic regions of *L. dumoffii dotA* (for gene deletion)	This study
pSR47SLpΔ*ravZ*	pSR47S containing 5' and 3' flanking genomic regions of *L. pneumophila ravZ* (for gene deletion)	This study
pMMB207	Cloning vector derived from RSF1010	[11]

pAM239	pMMB207 containing *gfp*mut3	[12]
pMMB207:: mCherry	pMMB207 containing *mCherry*	This study
pMMB207-*ravZ*	pMMB207 encoding *L. pneumophila ravZ*	This study
pMMB207-*ravZ* C258A	pMMB207 encoding *L. pneumophila ravZ* C258A	This study

Table S3. List of primers used in this study

pSR47SLdΔ*dotA*	*5'-* cg**gagctc**ATCAATACTACTCTCCATTA-3'	Δ *mutants*
	5'- AAAGAAATGAAAGTAGAATTGGTCGTGTTA-3'	
	5'- AATTCTACTTTCATTTCTTTTCTCCTAACA-3'	
	5'- gc**tctaga**CCACTACTTACTACTATAGT-3'	

pSR47SLdΔ*flaA*	5'-cg**gagctc**ACGAGCCGATATCCTGTGAT-3'	
	5'-TTTTCTATAAATTTTAGTCTCCTCAGACCT-3'	
	5'-AGACTAAAATTTATAGAAAAGATGTAAGA-3'	
	5'-gc**tctaga**AAAGCAGACAATTCCATAG-3'	
pSR47SLpΔ*ravZ*	5'-cgc**gagctc**CTCAACCCCTTTATTTAG-3'	
	5'-CAATTCACACTATTTGACCTCTAAACTG -3'	
	5'-AGAGGTCAAATAGTGTGAATTGAATTC -3'	
	5'-gc**tctaga**GGCTGACTGCAAAAGAGAGC -3'	
pSR47Sac1	5'-TAGCTCACTCATTAGGCACC-3'	Sequencing

pSR47SXba1	5'-CTGTTTCTTTTCATTCTCTG-3'	
GFPLC3 f	5′-AAAAAGCAGGCTATGGTGAGCAAGGGCGAG-3′	Cloning
GFPLC3 r	5′-AGAAAGCTGGGTGTGGTATGGCTGATTATGATC-3′	
Ulk1 f_2	5'-GCAAGGACTCTTCCTGTGACAC-3'	RT-PCR
Ulk1 r_2	5'-CCACTGCACATCAGGCTGTCTG-3'	
Ulk1 f	5'-AAGAACCTCGCCAAGTCTCA-3'	
Ulk1 r	5'-CCGTTGCAGTACTCCATAACC-3'	
GAPDH_f	5'-ACAGTCAGCCGCATCTTCTT-3'	
GAPDH_r	5'-ACGACCAAATCCGTTGACTC-3'	
GFP-Galectin8 f (BglII)	5'-ga**agatct**ATGATGTTGTCCTTAAACAACCTAC-3'	Cloning (pEGFP-C1)
GFP-Galectin8 r (SalI)	5′-ggccgac**gtcgac**CTACCAGCTCCTTACTTCCAGTAAG-3′	
GFP-NDP52 f (EcoRI)	5'-ccg**gaattc**ATGGAGGAGACCATCAAAGATCCC-3'	Cloning (pEGFP-C2)
GFP-NDP52 r (SalI)	5′-ggccgac**gtcgac**GAGAGAGTGGCAGAACACGTGGTC-3′	

ravZ f (BamHI)	5′-gc**ggatcc**AGAAAGGCAAGTTAACAGG-3′	Cloning (pMMB207)
ravZ r (XbaI)	5′-gc**tctaga**CTATTTTACCTTAATGCCACC-3′	
ravZ C258A f	5’-GAAGGCAATGCCGGGTCTTATACC-3’	Site-directed mutagenesis
ravZ C258A r	5’-GGTATAAGACCCGGCATTGCCTTC-3’	

References

1 Berger, K. H. & Isberg, R. R. Two distinct defects in intracellular growth complemented by a single genetic locus in Legionella pneumophila. *Mol Microbiol* **7**, 7-19 (1993).

2 Zuckman, D. M., Hung, J. B. & Roy, C. R. Pore-forming activity is not sufficient for Legionella pneumophila phagosome trafficking and intracellular growth. *Mol Microbiol* **32**, 990-1001 (1999).

3 Brenner, D. J. *et al.* Legionella-Bozemanii Sp-Nov and Legionella-Dumoffi Sp-Nov - Classification of 2 Additional Species of Legionella Associated with Human Pneumonia. *Curr Microbiol* **4**, 111-116 (1980).

4 Curtiss, R., 3rd & Kelly, S. M. Salmonella typhimurium deletion mutants lacking adenylate cyclase and cyclic AMP receptor protein are avirulent and immunogenic. *Infect Immun* **55**, 3035-3043 (1987).

5 Finan, T. M., Kunkel, B., De Vos, G. F. & Signer, E. R. Second symbiotic megaplasmid in Rhizobium meliloti carrying exopolysaccharide and thiamine synthesis genes. *J Bacteriol* **167**, 66-72 (1986).

6 Kabeya, Y. *et al.* LC3, a mammalian homologue of yeast Apg8p, is localized in autophagosome membranes after processing. *EMBO J* **19**, 5720-5728 (2000).

7 Ogawa, M. *et al.* A Tecpr1-dependent selective autophagy pathway targets bacterial pathogens. *Cell Host Microbe* **9**, 376-389 (2011).

8 Yoshikawa, Y., Ogawa, M., Hain, T., Chakraborty, T. & Sasakawa, C. Listeria monocytogenes ActA is a key player in evading autophagic recognition. *Autophagy* **5**, 1220-1221 (2009).

9 Takeshita, F. *et al.* TRAF4 acts as a silencer in TLR-mediated signaling through the association with TRAF6 and TRIF. *Eur J Immunol* **35**, 2477-2485 (2005).

10 Merriam, J. J., Mathur, R., Maxfield-Boumil, R. & Isberg, R. R. Analysis of the Legionella pneumophila fliI gene: intracellular growth of a defined mutant defective for flagellum biosynthesis. *Infect Immun* **65**, 2497-2501 (1997).

11 Morales, V. M., Backman, A. & Bagdasarian, M. A series of wide-host-range low-copy-number vectors that allow direct screening for recombinants. *Gene* **97**, 39-47 (1991).

12 Coers, J., Monahan, C. & Roy, C. R. Modulation of phagosome biogenesis by Legionella pneumophila creates an organelle permissive for intracellular growth. *Nat Cell Biol* **1**, 451-453 (1999).
